# UiO-type metal – organic frameworks as a platform for hydrogen storage studies

**DOI:** 10.1080/14686996.2026.2698228

**Published:** 2026-07-07

**Authors:** Osamu Oki, Takahiro Kondo

**Affiliations:** a Department of Materials Science, Institute of Pure and Applied Sciences, University of Tsukuba, Tsukuba, Japan; b Hydrogen Boride Research Center, Tsukuba Institute of Advanced Research (TIAR), University of Tsukuba, Tsukuba, Japan; c Tsukuba Research Center for Energy Materials Science (TREMS), University of Tsukuba, Tsukuba, Japan; d Research Center for Energy and Environmental Materials, National Institute for Materials Science, Tsukuba, Japan; e The Advanced Institute for Materials Research, Tohoku University, Sendai, Japan

**Keywords:** Hydrogen storage, metal–organic frameworks, UiO-type MOFs, physisorption, densification, hydrogen spillover, nanoconfinement

## Abstract

Hydrogen has attracted significant attention as a clean energy carrier due to its high gravimetric energy density and zero-carbon emissions at the point of use. However, its low volumetric energy density poses major challenges for safe and efficient storage. In this context, porous materials, particularly metal – organic frameworks (MOFs), have been extensively investigated as promising hydrogen-storage media because of their high surface areas, tunable pore environments, and structural diversity. Among them, Zr-based Universitetet i Oslo (UiO)-type MOFs have emerged as important platforms for hydrogen storage studies because of their exceptional framework stability, high synthetic versatility, and compatibility with the incorporation of functional species such as metal nanoparticles and hydrides. This review focuses on recent advances in hydrogen storage studies using UiO-type MOFs. First, the fundamental synthesis strategies and structural characteristics of UiO frameworks are outlined. Subsequently, key concepts of physisorption-based hydrogen storage, including adsorption mechanisms and evaluation metrics, are introduced. The hydrogen storage performance of pristine, functionalized, and densified UiO-type MOFs is then discussed, highlighting the influence of pore structure, linker functionality, and framework densification on hydrogen uptake. Beyond physisorption, chemisorption-related approaches, including spillover-assisted hydrogen storage and nanoconfinement of hydride species within UiO-type MOFs, are also highlighted. These studies demonstrate that UiO-type MOFs not only function as effective hydrogen adsorbents but also serve as versatile platforms for tuning hydrogen interactions. Finally, current challenges and future perspectives are discussed, providing insights into the development of next-generation hydrogen storage materials.

## IMPACT STATEMENT

This review highlights UiO-type MOFs as robust and tunable platforms for both physisorption- and chemisorption-related hydrogen storage, linking performance with functionalization, densification, spillover, and hydride nanoconfinement.

## GRAPHICAL ABSTRACT

**Figure:**
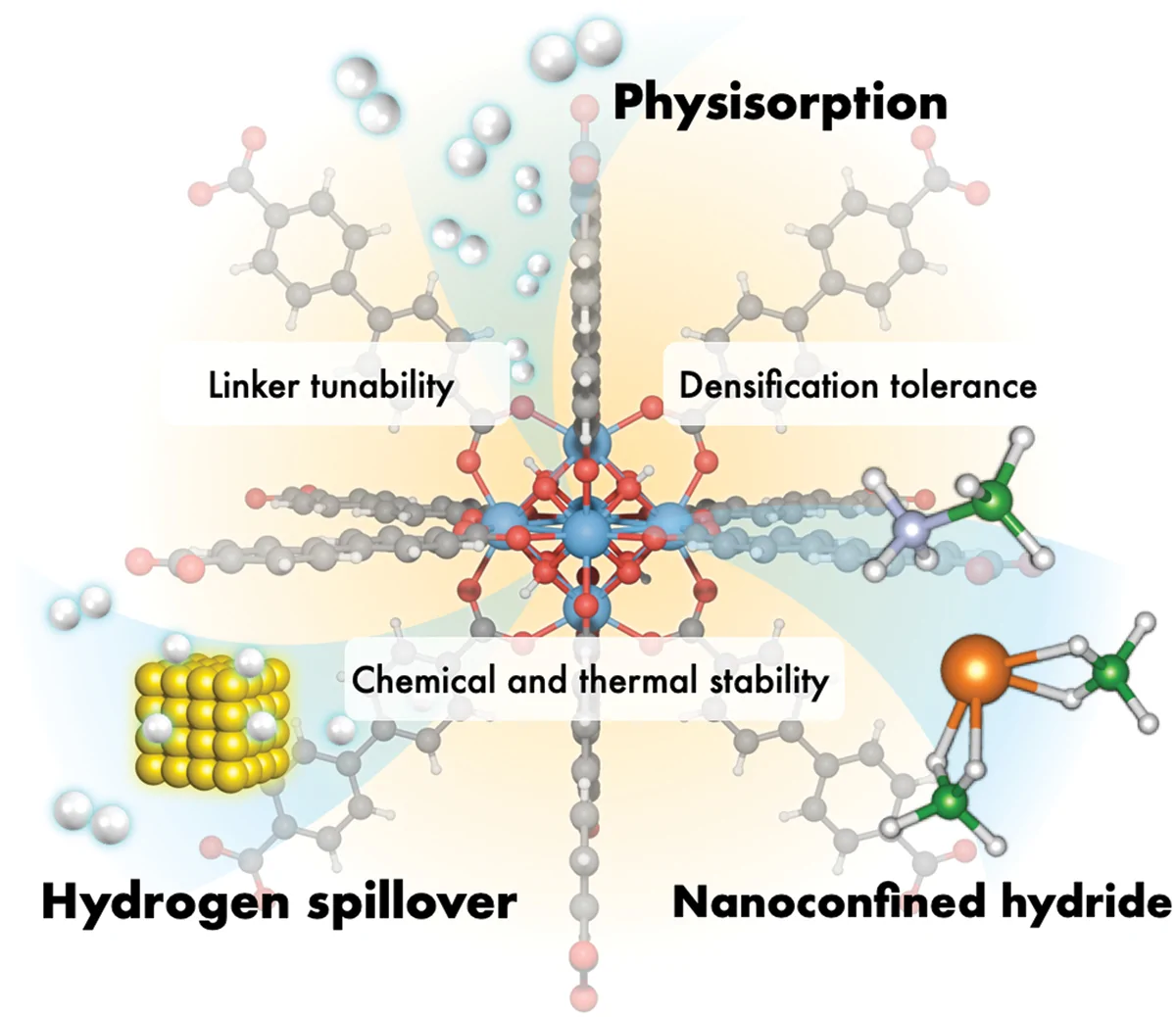
UiO-type MOFs as a versatile platform for hydrogen storage via physisorption, spillover, and nanoconfinement strategies.

## 1. Introduction

Hydrogen has attracted considerable attention as an environmentally friendly energy carrier because of its exceptionally high gravimetric energy density and absence of carbon dioxide emissions at the point of use [1–5]. On a mass basis, hydrogen stores approximately 120 MJ kg^−1^, which is nearly three times higher than gasoline (44 MJ kg^−1^) [1]. However, hydrogen has a very low volumetric energy density, making its storage and transportation challenging. Therefore, hydrogen is stored mainly as compressed gas or liquefied hydrogen. Compressed hydrogen storage requires high-pressure tanks typically operated at 35 or 70 MPa, whereas liquid hydrogen storage relies on cryogenic cooling to 20 K [6]. Although these technologies are already being implemented, both approaches have practical drawbacks, including the high energy cost of compression or liquefaction, strict requirements for storage and transport infrastructure, and safety concerns associated with high-pressure or cryogenic operations [4,7]. Therefore, extensive research has been devoted to developing alternative hydrogen storage technologies.

To overcome these limitations, alternative hydrogen-storage strategies based on solid-state materials have attracted increasing attention [8–11]. Among various hydrogen storage approaches, porous materials have been extensively studied because of their ability to accommodate hydrogen mainly through physisorption within internal pore structures, as well as through chemisorption- or spillover-related processes when metal species or catalytic components are incorporated [12–16]. In particular, metal – organic frameworks (MOFs) are regarded as one of the most versatile classes of porous materials [17–21]. Since the early 2000s, pioneering studies on MOF-5 [22], HKUST-1 [23], and related high-surface-area MOFs [24] have stimulated extensive research on hydrogen storage in MOFs. These early studies highlighted the importance of high surface area, pore structure, and accessible adsorption sites for hydrogen uptake. However, some early MOFs also suffered from limited chemical, thermal, or moisture stability, which restricted their use under practical and experimental conditions.

Against this background, Zr-based Universitetet i Oslo (UiO)-type frameworks have emerged as particularly attractive MOFs because of their exceptional chemical and thermal stability [25]. In addition to their robustness, their synthetic versatility and functional group tunability make UiO-type MOFs useful platforms for investigating structure – property relationships in various fields, including catalysis [26–35], photocatalysis [36–44], gas storage [45–55], water harvesting [56–59], water purification [60–63], membrane [64–68], D_2_/H_2_ separation [69], energy storage [70–73], chiral separation [74–76], uranium extraction [77–82], biomedical [83–87], and sensing [88–93] applications. For hydrogen storage, these characteristics make UiO-MOFs suitable for correlating synthetic design, framework stability, and hydrogen storage behavior. Therefore, UiO-MOFs should be regarded not simply as durable MOFs but also as versatile model systems for investigating structure – property relationships in hydrogen storage and guiding the rational design of next-generation solid-state hydrogen storage materials. To clarify the position of UiO-type MOFs within the broader landscape of hydrogen storage materials, Table 1 summarizes selected state-of-the-art solid-state and carrier-based hydrogen storage materials with different storage/release mechanisms [94–107].

**Table 1.** List of representative hydrogen storage materials.

Material class	Representative material	Main storage mechanism	Typical condition	Representative H_2_ capacity/uptake	Strength	Limitation	Ref
UiO-type MOF	UiO-66	Physisorption	77 K/high pressure	5.0 wt% (total), 29–74 g/L (total)	High chemical/thermal stability, tunable framework	Low room-temperature uptake	94
	UiO-67			4.8–5.4 wt% (total), 36–44 g/L (total)		Low room-temperature uptake	95
	UiO-68			–		Limited experimental H_2_-storage data	–
Benchmark MOFs	NU-1501-Al	Physisorption		14.5 wt% (total), 48 g/L (total)	High gravimetric and volumetric H_2_ uptake	Complex framework design	96
	MOF-177			6.3–7.5 wt% (total), 33–47 g/L (total)	High gravimetric uptake	Lower robustness than Zr-MOFs	95
	HKUST-1	Physisorption and open metal site interaction		5.7 wt% (total), 43 g/L (total)	Open Cu sites enhance H_2_ interaction	Moisture sensitivity and low-coverage saturation of open metal sites	97
	Mg-MOF-74			4.9 wt% (total), 45 g/L (total)	Open Mg sites enhance H_2_ interaction		98
Porous carbons	ZTC	Physisorption		6.0 wt% (total), 30–50 g/L (total)	High surface area, chemically robust carbon framework	Low room-temperature uptake	99
	C-PAF			17.49 wt% (total), 45.3 g/L (total)		Synthesis and processability issues	100
Metal hydride	MgH_2_	Reversible metal-hydride formation	Catalyst/nanostructure-assisted, moderate pressure	theor. 7.6 wt% (practical 4.5–6.7 wt%)	High gravimetric hydrogen capacity	High operating temperature and slow kinetics without modification	101
	LaNi_5_H_6_		Near room temperature/ moderate pressure	ca. 1.3–1.5 wt%	Reversible H_2_ absorption/desorption near room temperature	Low gravimetric capacity due to heavy alloy composition	102
Inorganic hydride	LiBH_4_	Chemical hydrogen release	Thermolysis/hydrolysis/catalytic dehydrogenation	theor. 18.5 wt% ca. 5–10 wt% reported	High theoretical gravimetric capacity	High dehydrogenation temperature and poor reversibility	103
	NH_3_BH_3_			theor. 19.6 wt%		Byproduct formation	104
	NH_3_ (liquid)	Catalytic decomposition	Catalytic cracking at elevated temperature	theor. 17.6 wt%	Carbon-free carrier; established production/transport infrastructure	Toxicity, corrosiveness, and need for NH_3_ cracking/removal	105
Liquid organic hydrogen carrier	MCH	Catalytic hydrogenation/dehydrogenation	Catalytic dehydrogenation at elevated temperature	theor. 6.1 wt%	Liquid at ambient conditions, compatible with existing fuel infrastructure	Cost and catalyst durability issues	106
	12 H-NEC			theor. 5.8 wt%		Cost and catalyst durability issues	107

In this review, we focus on UiO-type MOFs as a versatile platform for hydrogen storage studies rather than providing a broad comparison across different MOF families. We summarize recent progress in both physisorption- and chemisorption-related hydrogen storage approaches within the UiO-MOF family and discuss how structural design, linker functionalization, material densification, spillover, and nanoconfined hydrides influence hydrogen storage behavior. First, the fundamental synthetic strategies and structural characteristics of UiO-type frameworks are outlined. Second, we introduce the key parameters governing hydrogen storage through physisorption mechanisms, providing a foundation for evaluating storage capacity. We then review hydrogen physisorption studies of prototypical, functionalized, and densified UiO-type MOFs, focusing on the influence of structural modifications on their hydrogen storage performance. Third, we discuss key strategies for chemisorption-assisted hydrogen storage, highlighting the role of metal composites in enhancing storage capacity. Subsequently, recent developments in spillover-assisted hydrogen storage and the nanoconfinement of hydride species within UiO-type MOFs are explored. Finally, the current challenges and future perspectives for the design of UiO-based hydrogen storage materials are summarized. Through this platform-oriented perspective, this review aims to clarify how UiO-type MOFs can serve not only as hydrogen adsorbents but also as model systems for understanding structure – property relationships and guiding the rational design of solid-state hydrogen storage materials.

## 2. Synthesis and structural features of UiO-type MOFs

The synthesis chemistry of MOFs plays a central role in determining their crystallinity, structural stability, defect formation, and adsorption-related properties. Therefore, before discussing hydrogen storage in detail, it is useful to briefly outline the fundamental synthetic strategies for UiO-type MOFs as a basis for understanding their structural features. Detailed discussions on the synthesis of UiO-type MOFs can be found in several comprehensive reviews [108,109]. Herein, we briefly summarize the key aspects of their synthesis.

### 2.1. Synthesis of prototypical UiO-type MOFs

**Figure 1.**
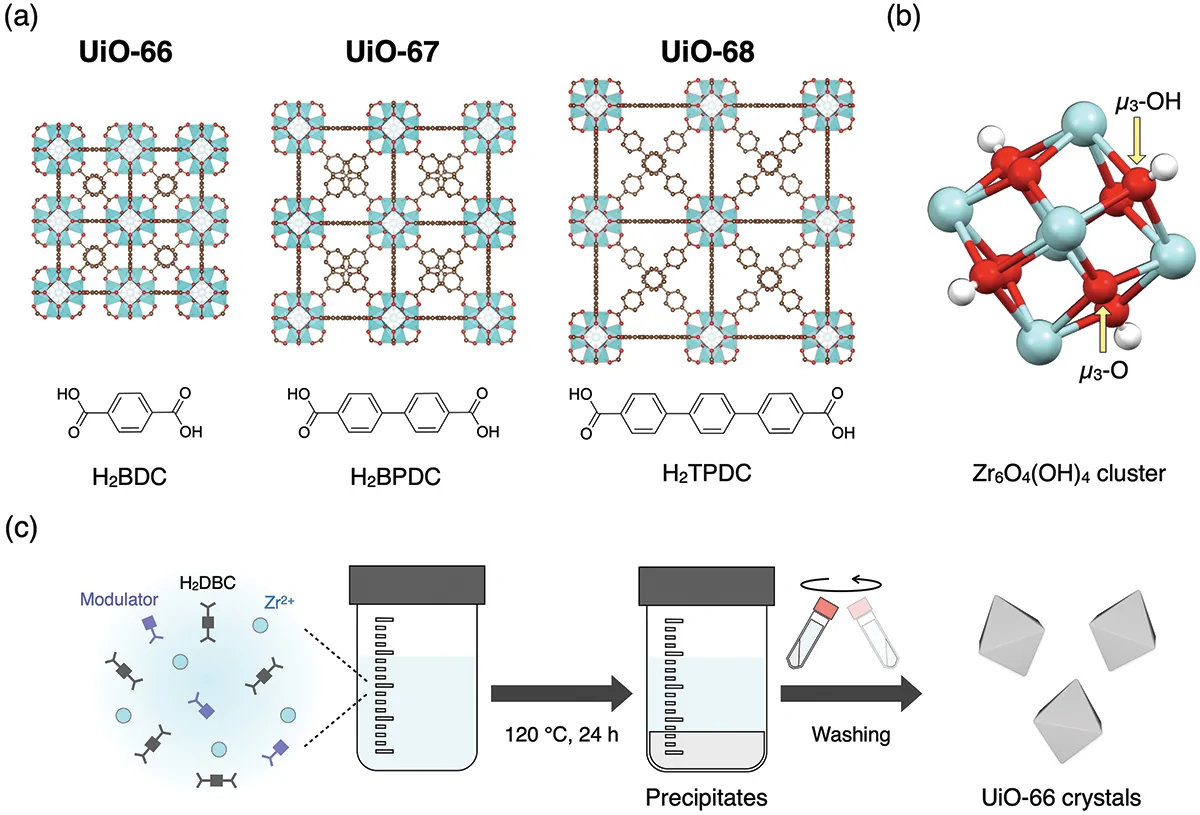
(a) Framework structures of UiO-66, UiO-67, and UiO-68 together with the corresponding organic linkers H_2_BDC, H_2_BPDC, and H_2_TPDC, respectively. In panel (a), the framework is shown using polyhedral and ball-and-stick representations, where cyan, red, and brown denote Zr, O, and C atoms, respectively. Hydrogen atoms are omitted for clarity. (b) Schematic structure of a Zr_6_O_4_(OH)_4_ cluster. The *μ*_3_-OH and *μ*_3_-O groups indicate that each hydroxo or oxo group bridges three Zr atoms. In panel (b), cyan, red, and white spheres represent Zr, O, and H atoms, respectively. (c) Schematic representation of a typical synthesis procedure of UiO-66. Monocarboxylic acids such as benzoic acid and acetic acid are often used as a modulator.

Cavka et al. [25] first reported the solvothermal synthesis of prototypical UiO-66, UiO-67, and UiO-68 compounds in 2008 (Figure 1(a)) [25]. These UiO-type frameworks are constructed from Zr_6_O_4_(OH)_4_ clusters and linear dicarboxylate linkers. In the Zr_6_O_4_(OH)_4_ cluster, six Zr atoms form an octahedral core, with the triangular faces of the Zr_6_ octahedron capped by *µ*_3_-OH or *µ*_3_-O groups, where *µ*_3_ indicates that each oxo or hydroxo group bridges three Zr atoms (Figure 1(b)). These frameworks are typically prepared by reacting Zr precursors with dicarboxylate linkers in polar aprotic solvents. For instance, UiO-66 was synthesized from ZrCl_4_ and terephthalic acid (H_2_BDC) in *N,N*-dimethylformamide (DMF) containing a controlled amount of water under solvothermal conditions, typically with a reaction time of around 24 h (Figure 1(c)). During solvothermal heating, the Zr precursor undergoes hydrolysis and condensation to generate the Zr_6_O_4_(OH)_4_ clusters, followed by coordination with terephthalate linkers to form the UiO-66 framework. Similarly, the authors synthesized a series of isoreticular frameworks with increasing linker lengths, including UiO-67 (4,4′-biphenyldicarboxylic acid (H_2_BPDC)) and UiO-68 (4,4″-terphenyldicarboxylic acid (H_2_TPDC)). The exceptional chemical and thermal stabilities of UiO-type MOFs are generally attributed to the highly connected Zr_6_-based framework topology, together with the strong Zr–O bonds [110–114]. The bond dissociation energy of the Zr–O bond is estimated to be approximately 760 kJ mol^−1^, which is significantly higher than those of the Al–O (~512 kJ mol^−1^), Cu–O (~343 kJ mol^−1^), and Zn–O (~284 kJ mol^−1^) bonds found in representative MOFs [115], such as MIL-100 (Al–O bond), HKUST-1 (Cu–O bond), and MOF-5 (Zn–O bond). Furthermore, Zr_6_O_4_(OH)_4_ features a 12-coordinated node, which imparts high structural stability. These characteristics are reflected in the remarkable thermal stability of UiO-66 (475 °C), which is significantly higher than those of other common MOFs such as Al-MIL-100 (400 °C) and HKUST-1 (300 °C) [116]. UiO-66 also exhibits exceptional mechanical stability, with a minimum shear modulus of 13.7 GPa, significantly higher than those of other benchmark MOFs, including MOF-5 (1.4 GPa) and HKUST-1 (1.04 GPa) [117]. These characteristics make UiO-type MOFs an outstanding platform for a wide range of hydrogen-related studies. Compared with UiO-66 and UiO-67, pristine UiO-68 has been investigated less extensively. This may partly reflect the limited solubility of H_2_TPDC in common organic solvents, which makes its synthesis more sensitive to the reaction conditions. Nevertheless, UiO-68-type frameworks can be obtained more readily when functionalized linkers are employed [118,119].

In addition to typical solvothermal synthesis, alternative synthetic strategies, including microwave-assisted synthesis [120–123], aqueous solution-based production [124,125], mechanochemical synthesis [126–129], and flow synthesis [130–133] have also been explored for UiO-type MOFs. These methods have considerable potential to reduce production costs and improve scalability. Based on laboratory-scale solvothermal synthesis, the material cost of UiO-66 has been estimated to be approximately 503.9 USD kg^−1^ [134]. This cost is comparable to, or even lower than, those of other widely studied MOFs, such as MOF-5 (527.3 USD kg^−1^) and Ni-MOF-74 (886.7 USD kg^−1^), indicating that UiO-type MOFs are already cost-competitive for laboratory-scale studies. However, further cost reduction is necessary for practical applications to achieve target production costs as low as 10–15 USD kg^−1^ [135,136]. In this context, a techno-economic assessment based on hypothetical pilot-scale production of amine-functionalized UiO-66 suggested that aqueous solution-based synthesis could reduce the production cost to approximately 15.8 USD kg^−1^ [137]. This result indicates that substantial cost reductions may be achievable for UiO-type MOFs through solvent replacement, improved yields, and process scale-up.

### 2.2. Acid-modulated synthesis and defect engineering

The crystallinity and structural defects of MOFs are strongly related to their gas sorption applications [138–140]. From the first report of the prototypical UiO series, the addition of a modulator in the synthesis has been shown to improve the reproducibility and crystallinity, as well as being useful for controlling the particle size and density of defects [141,142]. Representative studies on the acid-modulated synthesis and defect engineering of UiO-type MOFs are summarized below.

#### 2.2.1. Acid modulation

Early synthetic studies on UiO-type MOFs revealed that achieving highly crystalline products is not always straightforward. In 2011, Schaate et al. [143] reported that although the original procedure reproducibly produced highly crystalline UiO-66, the synthesis of UiO-67 often led to poorly crystalline products, and attempts to synthesize UiO-68 were unsuccessful [143]. They found that acid-modulated synthesis using monocarboxylic acids such as benzoic acid and acetic acid markedly improved the crystallinity and reproducibility of UiO-66 and UiO-67, while also enabling control over the crystal size over a wide range, from nanosized particles to single crystals. The mechanism was proposed to involve the competitive coordination of monocarboxylic acids with the dicarboxylate linker at the Zr_6_ clusters, thereby slowing nucleation and crystal growth and improving crystallinity and reproducibility.

**Figure 2.**
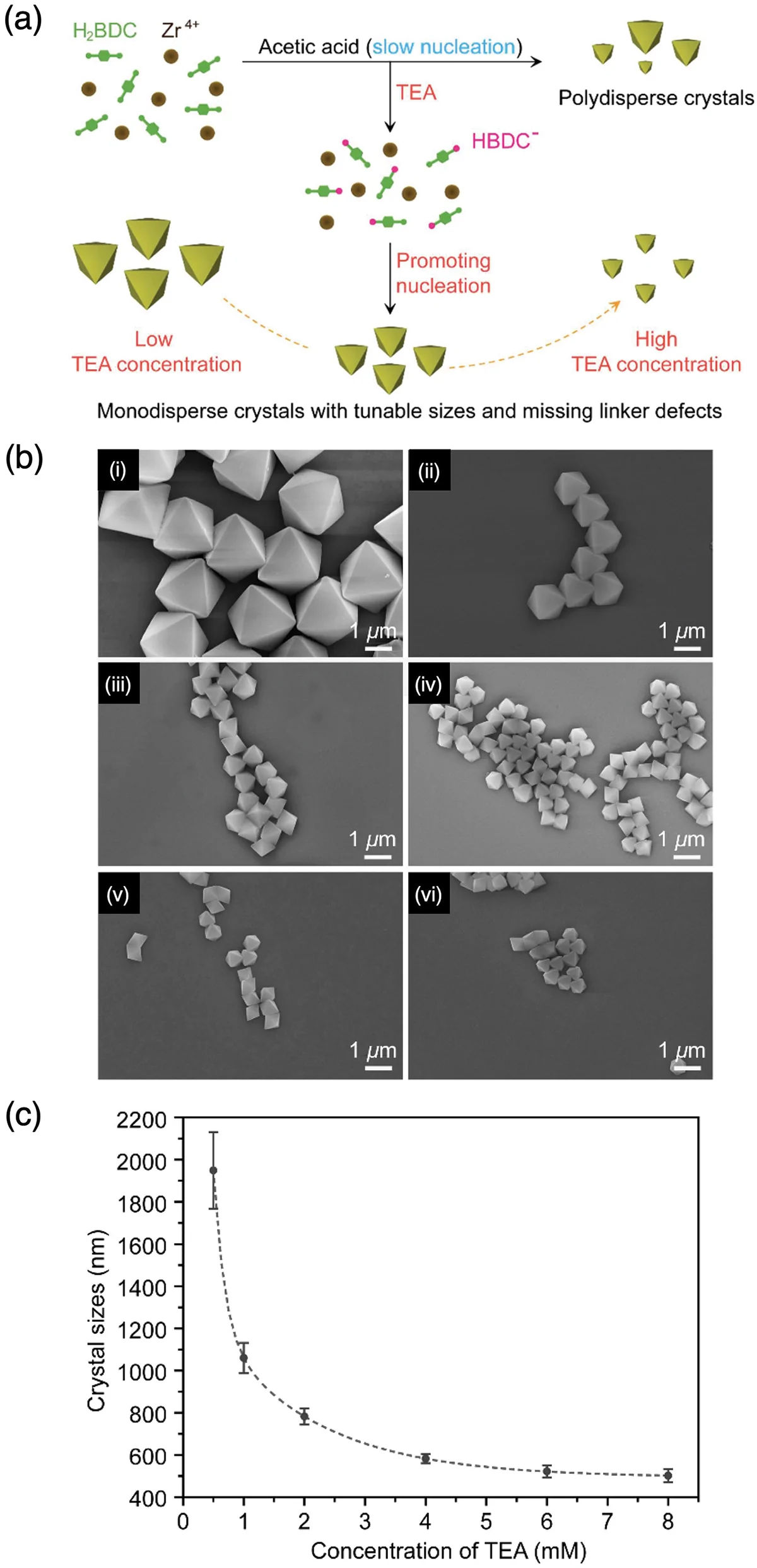
(a) Illustration of the acetic acid-modulated and acetic acid/triethylamine (TEA) comodulated syntheses of UiO-66 in large scale. (b) Scanning electron microscopy (SEM) images of UiO-66 crystals synthesized in the presence of 4 mM ZrCl_4_, 4 mM H_2_BDC, 2.4 M acetic acid, and TEA at concentrations of (i) 0.5, (ii) 1, (iii) 2, (iv) 4, (v) 6, and (vi) 8 mM, respectively. (c) Statistical average crystal sizes of samples obtained from four independent batches of reactions with the same experimental parameters versus TEA concentration. Reproduced with permission [149] 2017, American Chemical Society.

Since then, the use of acid modulators has become a widely adopted strategy in the synthesis of UiO-type MOFs for controlling crystallization behavior [144–148]. Zhao et al. [149] further developed an acid/base co-modulation strategy in which acetic acid and triethylamine (TEA) were used to control crystal growth and nucleation, enabling the large-scale synthesis of monodisperse UiO-66 crystals with tunable sizes and defect densities (Figure 2) [149]. In terms of structural defects, modulation has also been recognized as strongly related to defect formation in UiO-type frameworks, as discussed in Section 2.2.2.

#### 2.2.2. Structural defects

**Figure 3.**
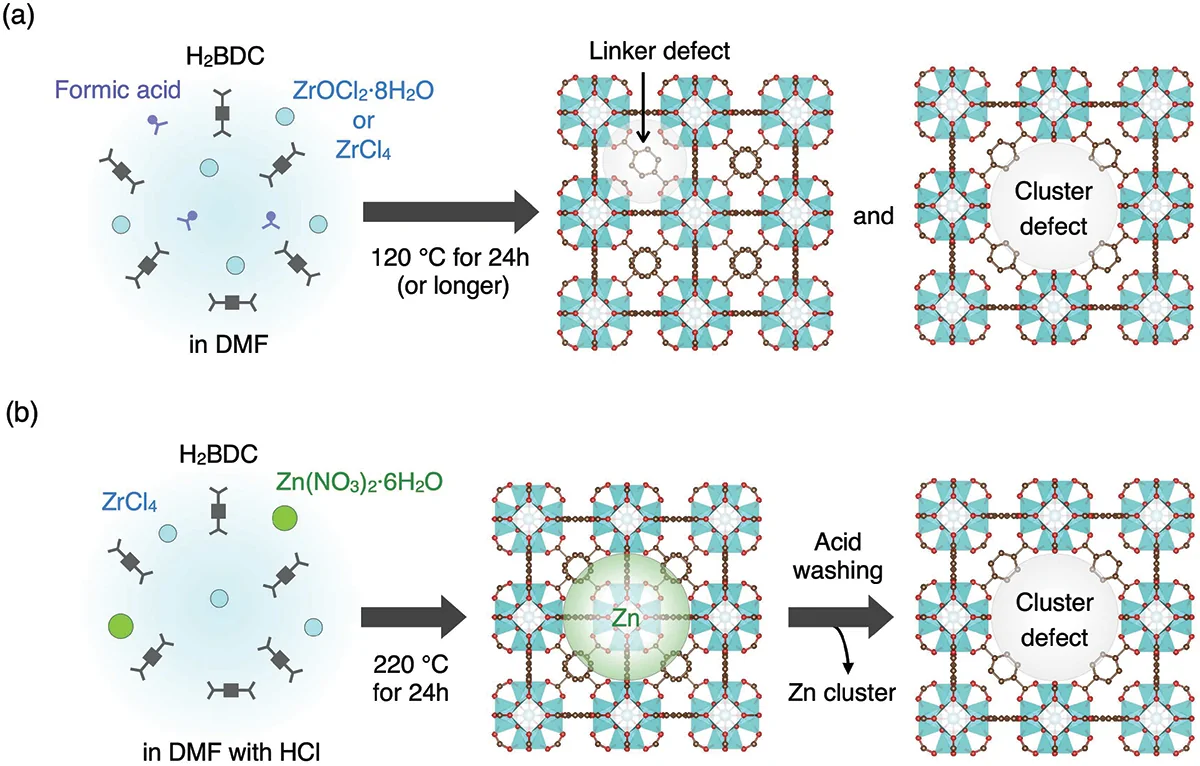
Schematic illustration of defect formation strategies in UiO-66. (a) Formic-acid-modulated synthesis of UiO-66, which can generate missing-linker and/or missing-cluster defects. (b) Post-synthetic creation of selective missing-cluster defects in mixed-metal (Zn, Zr)-UiO-66 by selective removal of acid-labile Zn species. The mixed-metal UiO-66 is first synthesized using a Zn precursor and subsequently treated by acid washing, resulting in missing-cluster-defective UiO-66. Cyan, red, gray, and white spheres represent Zr, O, C, and H atoms, respectively.

Structural defects have attracted considerable attention in UiO-type MOFs because they can significantly alter the local structure and thus influence the physicochemical properties of the framework [150–152]. Valenzano et al. [153] first reported missing linker defects in UiO-66 based on thermogravimetric analysis [153]. Their analysis suggested that approximately one in 12 terephthalate linkers coordinated to each Zr_6_ node was missing. Subsequently, Wu et al. [154] provided the first direct structural evidence of missing linker defects in UiO-66 using high-resolution neutron diffraction [154]. They demonstrated that the concentration of missing-linker defects could be systematically tuned by varying the amount of acetic acid used as a modulator and the synthesis time. Gutov et al. [155] reported that formic acid modulation introduced modulator-capped defects in UiO-67, which were primarily discussed in the context of missing-linker defects [155]. However, Shearer et al. [156] later showed that, under typical monocarboxylic-acid-modulated synthesis conditions, defective UiO-66 samples are often dominated by missing-cluster defects [156] (Figure 3(a)). Furthermore, Bueken et al. [157] developed a mixed-linker strategy to selectively introduce missing-linker defects in UiO-66 [157]. They incorporated a thermally labile linker, trans-1,4-cyclohexane-dicarboxylate, together with the conventional terephthalate linker during UiO-66 synthesis. Upon subsequent thermolysis, the labile linker was selectively removed from the framework, generating missing-linker defects without relying on conventional modulator-induced defect formation. In contrast to these approaches for generating missing-linker defects, Feng et al. [158] reported a post-synthetic strategy for creating missing-cluster defects in UiO-66 [158]. They first synthesized bimetallic (Zn, Zr)-UiO-66 samples with different Zn/Zr ratios and then applied a mild acid-washing treatment (Figure 3(b)). Because Zn nodes are less stable than Zr nodes under acidic conditions, the Zn species could be selectively removed while preserving the UiO-66 framework, resulting in well-defined missing-cluster defects.

Separately, using high-resolution transmission electron microscopy (HRTEM), atomic-scale visualization studies have provided further insight into the spatial arrangement of defects in UiO-type frameworks [159,160]. For example, Liu et al. directly visualized ordered missing-linker and missing-cluster defects in UiO-66 at sub-unit-cell resolution. In their study, defective UiO-66 was synthesized using formic acid as a modulator, and HRTEM revealed that ordered missing-linker regions were widely distributed, whereas missing-cluster defects appeared as smaller nanoscale domains. Three-dimensional reconstruction further showed that the missing-linker structure consisted of 8-connected Zr_6_ clusters capped by terminal formate ligands, providing direct real-space evidence for the local ordering and termination of linker vacancies [159].

A broader overview of defect engineering in UiO-66, including various types of defects and their formation, characterization, and effects on the physicochemical properties, has been comprehensively summarized in another review [161,162]. Because hydrogen adsorption in porous materials strongly depends on the accessible surface area and pore structure, defect engineering in UiO-type frameworks has attracted considerable attention as a strategy for tuning adsorption properties.

### 2.3. Structural diversity in functionalized UiO-type MOFs

In addition to defect engineering, another important strategy for tuning the properties of UiO-type MOFs is the introduction of functional groups into the organic linkers. Because the UiO framework can accommodate a wide variety of dicarboxylate ligands while maintaining the same Zr-cluster node and topology, numerous functionalized derivatives have been developed [109,118,119,163,164]. Such functionalization can significantly alter the pore environment, including the pore size, polarity, and distribution of adsorption sites. Consequently, functionalized UiO-type MOFs exhibit considerable structural diversity and offer opportunities to tailor their physicochemical properties.

An early example is UiO-66-NH_2_, in which amino-substituted terephthalate linkers are incorporated while preserving the UiO-66 framework [165]. The presence of amino groups provides additional chemical functionality, making this material an important platform for catalysis and post-synthetic modification [28,38–41,43,44,51,58,62,66,67,76,81,83,90]. Another important class is the pyridine-containing UiO-67 derivatives, such as frameworks constructed from bipyridine-based dicarboxylate linkers. These materials introduce coordination-capable nitrogen sites into the framework and are, therefore, useful for further metalation and catalytic applications [30,68,164]. Furthermore, variations in the organic linker allow reproducible synthetic access to members of the UiO-68 family [118,119], even though the pristine UiO-68 framework is comparatively difficult to prepare reproducibly. Importantly, these functionalization strategies have been explored in the context of hydrogen storage. Their specific roles in tuning the hydrogen adsorption behavior and expanding the design versatility of UiO-type frameworks are discussed in Section 3.

## 3. UiO-type MOFs as hydrogen adsorption media

Owing to their high chemical and thermal stabilities, structural tunability, and synthetic versatility, UiO-type MOFs represent one of the most extensively studied Zr-based MOF families. These characteristics render these materials useful platforms for systematically elucidating the design principles of physisorption-based H_2_ storage. In this section, we first summarize the fundamental parameters relevant to physisorption-based hydrogen storage and then review previous studies on H_2_ storage in prototypical, functionalized, and densified UiO-type MOFs.

## 3.1. Fundamental parameters for physisorption-based hydrogen storage

Before discussing hydrogen storage studies on UiO-type MOFs, this section briefly summarizes the fundamental concepts, terminology, and evaluation parameters relevant to physisorption-based hydrogen storage. To provide a consistent basis for the following discussion, the definitions and evaluation criteria are outlined below.

### 3.1.1. Physisorption

Hydrogen storage in porous materials typically relies on physisorption [166,167]. Physisorption refers to the adsorption of molecules onto a material surface via weak intermolecular interactions, primarily van der Waals forces. In general, physisorption does not significantly alter the chemical structures of either the adsorbate or the adsorbent. In the case of H_2_, owing to its relatively weak interaction strength, typically corresponding to an adsorption enthalpy (Δ*H*) of around −4 to −7 kJ mol^−1^, physisorption enables reversible H_2_ adsorption and desorption through temperature and pressure swings. In other words, the intrinsically weak H_2_···adsorbent interaction requires rational material design to enhance hydrogen storage performance through strategies such as tuning the pore size, increasing the surface area, and optimizing the adsorption enthalpy.

We note that although pressures of 35–70 MPa are typically employed in practical hydrogen storage systems, a pressure range of 10–20 MPa is commonly used for high-pressure hydrogen adsorption measurements in laboratory-scale studies. This range is often adopted to benchmark the performance of porous materials because it is compatible with the upper pressure limits of commercially available hydrogen storage tanks. Achieving higher pressures requires additional compression equipment and more stringent safety protocols. Thus, this pressure range serves primarily as a reference condition for experimental evaluation rather than as a direct target for practical hydrogen storage applications.

### 3.1.2. Excess and total H_2_ uptake

**Figure 4.**
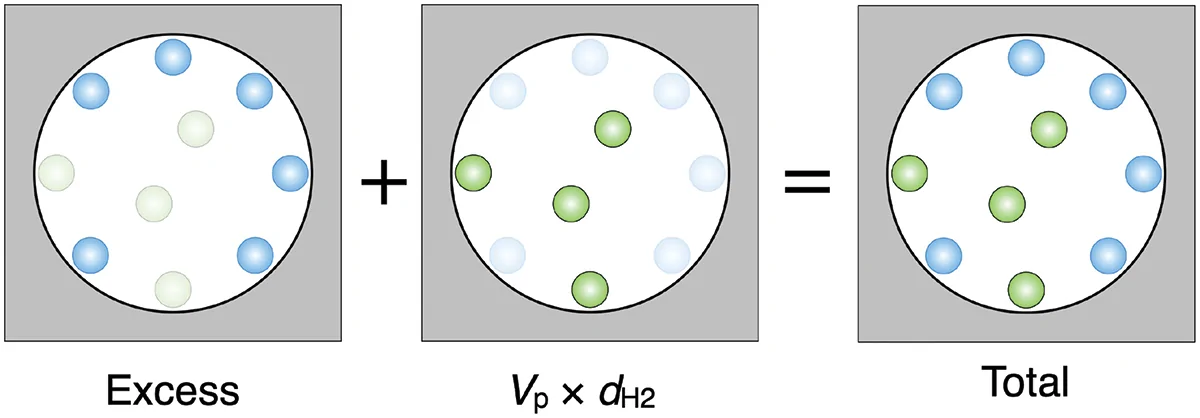
Schematic of the relationship between excess and total hydrogen adsorption in porous materials. The total uptake is expressed as the sum of the experimentally measured excess adsorption (shown as blue balls) and the contribution from the bulk gas (shown as green balls) within the pore volume (*V*_p_ × *d*_H2_).

The amount of hydrogen stored in adsorbents is generally evaluated in terms of excess and total adsorption. Excess adsorption represents the amount of hydrogen present in excess of that in the free gas phase within the same volume, whereas the total hydrogen uptake includes both the excess adsorbed H_2_ and compressed H_2_ gas present in the pores (Figure 4). Experimentally, H_2_ adsorption is typically evaluated using volumetric or gravimetric pressure – composition – temperature (PCT) measurements. In the volumetric method, the amount of adsorbed H_2_ is calculated from the gas pressure drop in the measurement system using a gas-state equation, whereas the gravimetric method is based on directly monitoring the weight change of a sample upon gas adsorption using a highly sensitive microbalance.

In these measurements, the directly obtained values correspond to the excess gravimetric H_2_ adsorption (*N*_ex_) (e.g. gram H_2_ per gram of adsorbent). The total gravimetric H_2_ uptake, *N*_tot_, is calculated by accounting for the contribution of compressed gas to the pore volume (*V*_p_) (Eq. 1).

(1) $$N_{\mathrm{tot}}=N_{\mathrm{ex}}+d_{H2}\times V_{p}$$

Here, *d*_H2_ (g cm^−3^) is the density of compressed H_2_ at a given temperature and pressure. *V*_p_ (cm^3^ g^−1^) is generally determined from N_2_ sorption isotherms at 77 K, usually considering the amount of N_2_ adsorbed at *P*/*P*_0_ ≈ 0.95 based on the Gurvich rule. However, this value should be interpreted carefully because capillary condensation in the interparticle macropores can lead to an overestimation of *V*_p_. The volumetric H_2_ uptake (e.g. grams of H_2_ per liter of adsorbent) can be estimated from the gravimetric uptake using the adsorbent density. Because this conversion is highly sensitive to the definition of density, including the crystal, bulk, and tap densities, volumetric capacities should be treated with caution.

**Figure 5.**
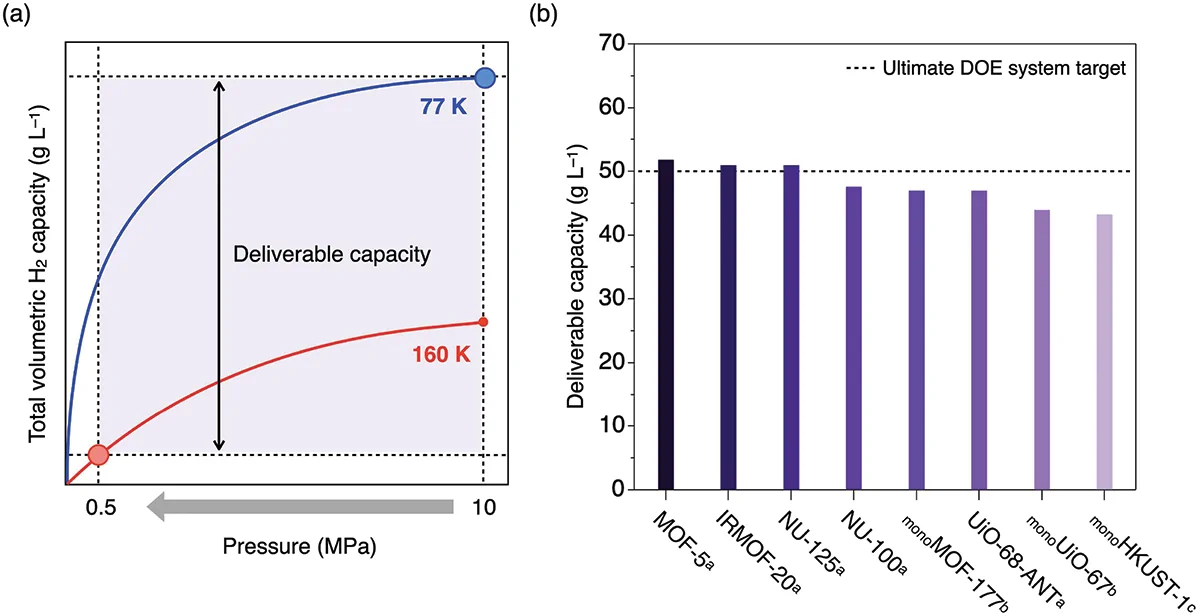
(a) Illustration of total volumetric hydrogen uptake as a function of pressure at 77 K (blue curve) and 160 K (red curve), showing the concept of deliverable capacity. (b) Comparison of volumetric deliverable H_2_ capacities for various MOFs under defined pressure- and temperature-swing conditions, with the dashed line indicating the DOE system-level target. These data were collected from the different literatures [19,95,97,169]. Superscripts next to the MOF names indicate the method used for volumetric calculation: ^a^calculated based on packing density, ^b^calculated based on crystallographic density, ^c^calculated based on envelope density obtained from mercury intrusion porosimetry.

In the field of MOF-based hydrogen storage, deliverable capacity in terms of both gravimetric and volumetric performance is widely regarded as the most important metric. The U.S. Department of Energy (DOE) has established system-level targets of 5.5 wt% (40 g L^−1^) for 2025 and an ultimate target of 6.5 wt% (50 g L^−1^) for onboard H_2_ storage systems. These targets include the weight contributions of the storage tank, storage material, and auxiliary system components [168]. Direct comparison should therefore be interpreted cautiously. Although MOF powders alone do not represent complete storage systems, material-level capacities that approach or exceed these system-level targets are generally considered strong indicators of promising adsorbents. High-performing MOFs often achieve gravimetric deliverable capacities of 5–9 wt% and volumetric deliverable capacities of 40–50 g L^−1^ under defined pressure- and temperature-swing conditions (e.g. 10 MPa at 77 K to 0.5 MPa at 160 K) [19,95,97,168,169], highlighting their potential to contribute significantly toward practical hydrogen storage applications (Figure 5).

### 3.1.3. Specific surface area

**Figure 6.**
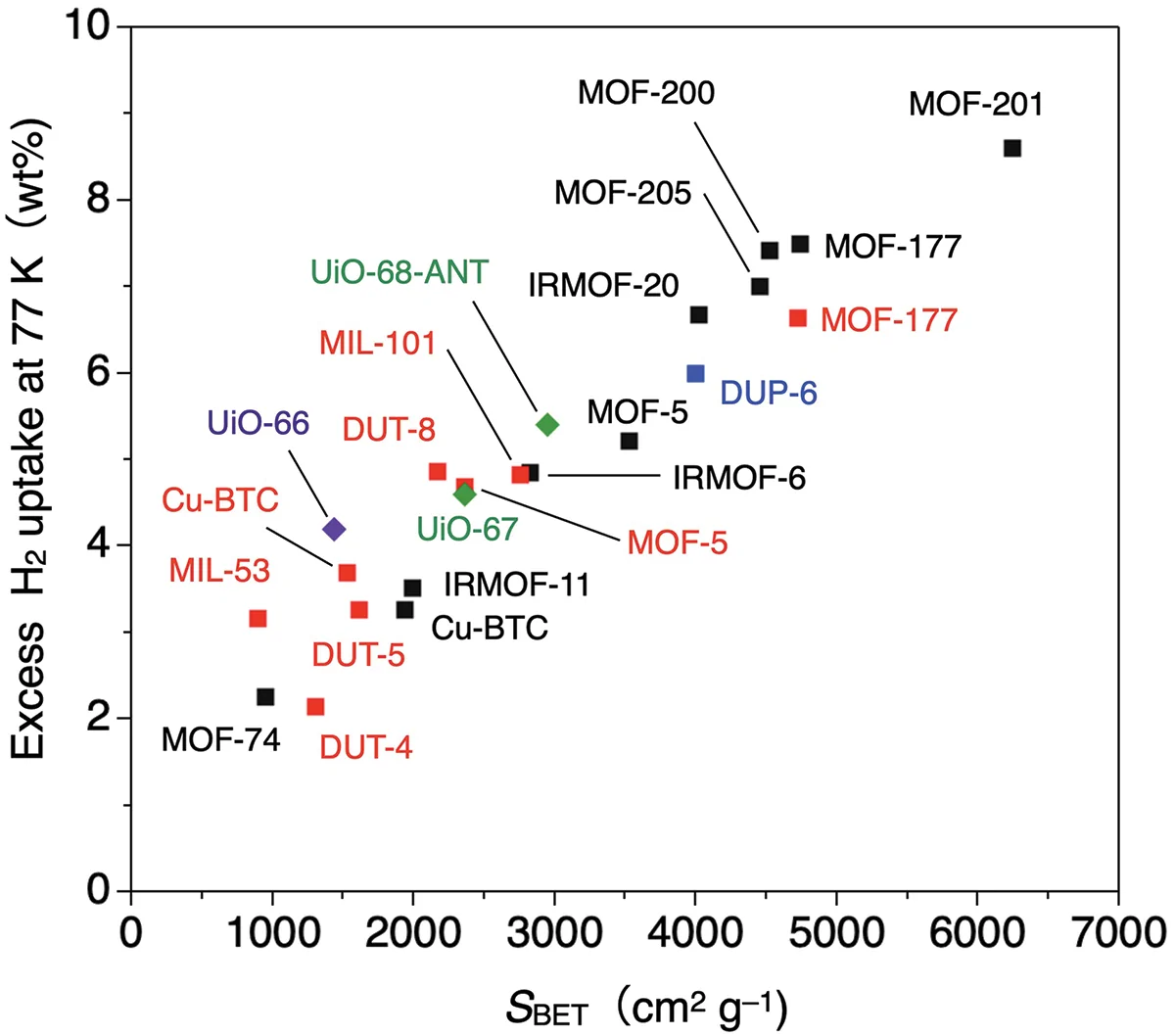
A plot of excess gravimetric H_2_ uptake, measured at 77 K and 2 MPa or above, for various MOFs. The data points of UiO-66 were taken from ref. [170], while those of UiO-67 and UiO-68-ANT were taken from ref. [97], respectively. All other data were approximated from the plot in ref. [171] 2011, WILEY. Colors denote measurements conducted by different research groups.

Because physisorption occurs on the internal surfaces of porous materials, the specific surface area is a major parameter that governs the hydrogen storage capacity. The empirical correlation between cryogenic gravimetric H_2_ uptake and specific surface area is commonly referred to as the Chahine rule (i.e. 1 wt% excess H_2_ uptake for every 500 m^2^ g^−1^ of surface area) (Figure 6) [171]. At ambient temperatures, this relationship becomes less universal, as H_2_ uptake depends more strongly on additional factors such as adsorption enthalpy, pore size, and the nature of the adsorption sites. A comprehensive discussion of the correlation between the surface area and H_2_ uptake can be found in previous reviews [18].

The specific surface areas of porous materials are generally determined from the N_2_ adsorption isotherms measured at 77 K. Because the calculated surface area depends on the adsorption model used for the analysis, the obtained values are typically expressed as either Brunauer – Emmett – Teller (BET) [172–174] or Langmuir surface areas [175,176]. In hydrogen storage studies of MOFs, the BET surface area (*S*_BET_), which takes into account multilayer adsorption on the surface, is more commonly used as the standard descriptor. This is because the Langmuir model, which is based on an ideal monolayer adsorption assumption, often overestimates the surface area of microporous adsorbents.

### 3.2. H_2_ storage in prototypical UiO-MOFs

**Figure 7.**
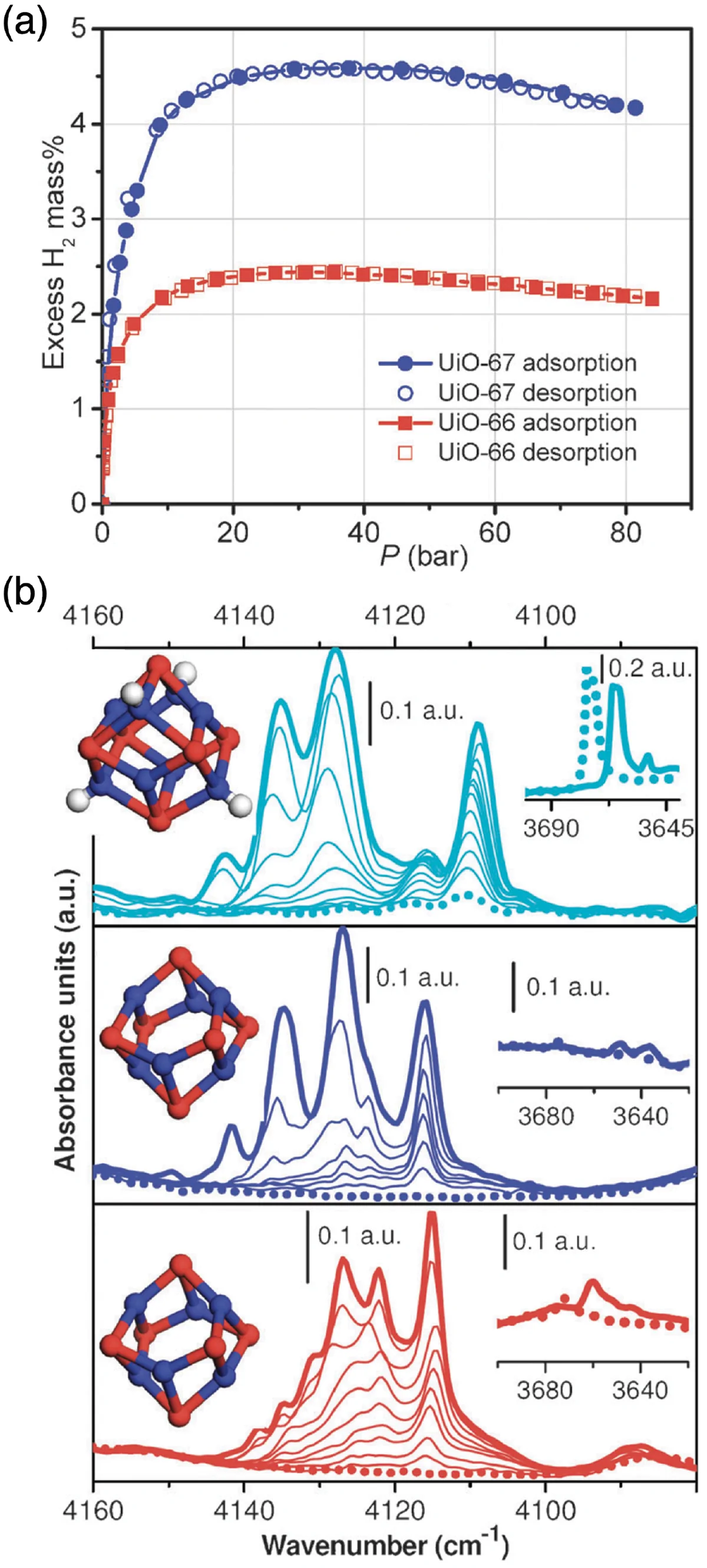
(a) H_2_ excess isotherms recorded at 77 K on UiO-66 (red squares) and UiO-67 (blue circles). Filled and open symbols refer to the adsorption and desorption branches, respectively. (b) VT-FTIR spectra of H_2_ adsorbed on hydroxylated UiO-67 (top panel), dehydroxylated UiO-67 (middle panel), and dehydroxylated UiO-66 (bottom panel) as a function of temperature (100–115 K). Dotted curves represent the activated material before H_2_ dosage (spectra collected at 15 K). Bold curves represent the highest H_2_ coverage (spectra collected at 15 K). Intermediate curves, from bold to dotted curves, represent spectra collected at increasing temperatures. Right insets show the *ν*(OH) stretching region of the IR spectra before (dotted curves) and after (bold curves) H_2_ dosage. In the structural models, red, blue, and white spheres represent Zr, O, and H atoms, respectively. Reproduced with permission [177] 2012, Royal society of chemistry.

In 2012, Chavan et al. [177] first reported high-pressure H_2_ adsorption using UiO-66 and UiO-67 [177]. UiO-66 and UiO-67 were synthesized using a typical solvothermal method in the presence of a small amount of water (95°C for 100 h). N_2_ sorption measurements at 77 K showed that UiO-66 and UiO-67 exhibited *S*_BET_ values of 969 and 1877 m^2^ g^−1^, respectively, with corresponding *V*_p_ values of 0.43 and 0.85 cm^3^ g^−1^. The H_2_ adsorption isotherms at 77 K showed fully reversible behavior for both UiO-66 and UiO-67, as shown in Figure 7(a). The maximum gravimetric excess uptake reached 2.4 wt% at 0.32 MPa for UiO-66 and 4.6 wt% at 0.38 MPa for UiO-67. Although the H_2_ uptake of UiO-67 was among the highest reported at that time, its superior chemical and thermal stability highlighted its potential advantages.

Variable-temperature Fourier transform infrared (VT-FTIR) spectroscopy was conducted (15–300 K under an H_2_ pressure of 4.5–5.0 × 10^−3^ MPa) to elucidate the nature of the H_2_ adsorption sites (Figure 7(b)). Notably, free H_2_ is essentially infrared (IR)-inactive and, therefore, is not directly observable in conventional IR spectroscopy. By contrast, H_2_ molecules become polarized upon adsorption on cationic or anionic surface sites, making the *v*(H–H) stretching vibrational mode IR-active. The *v*(H–H) bands appeared in the temperature range of 100–115 K and were red-shifted from the gas-phase Raman value (4160 cm^−1^). For band assignment, the VT-FTIR spectra of hydroxylated UiO-67 and its dehydroxylated counterpart were compared, with dehydroxylated UiO-66 used as a reference. The most strongly perturbed component was located at 4108 cm^−1^ (Δ*v* = −52 cm^−1^), which was assigned to H_2_ interacting with the *µ*_3_-OH groups of the Zr_6_O_4_(OH)_4_ node as this band markedly decreased upon dehydroxylation. By contrast, a band at 4116 cm^−1^ (Δ*v* = −44 cm^−1^) became dominant after dehydroxylation, suggesting adsorption on *µ*_3_-O sites. At higher coverage, additional features in the 4120–4145 cm^−1^ region were observed, which were associated with interactions with the organic linker and/or condensed H_2_ in the pores. These assignments suggest that preserving the *µ*_3_-OH sites is beneficial for H_2_ adsorption.

At around the same time, Abid et al. [170] reported nanosized UiO-66 as a hydrogen adsorbent after optimizing the activation procedure through solvent exchange, yielding an *S*_BET_ of 1434 m^2^ g^−1^ and a H_2_ uptake of 4.2 wt% at 77 K and 6.0 MPa [170]. The authors also evaluated the isosteric heat of adsorption (*Q*_st_) from temperature-dependent H_2_ isotherms. The *Q*_st_ value was 11.9 kJ mol^−1^ at low loading and gradually decreased to 5.8 kJ mol^−1^ with increasing H_2_ surface coverage, indicating a typical physisorption behavior. The initial value of 11.9 kJ mol^−1^ indicates relatively strong H_2_–framework interactions and approaches the range often considered favorable for adsorption-based H_2_ storage (approximately 10–15 kJ mol^−1^ or higher [178]). However, the decrease to 5.8 kJ mol^−1^ with increasing H_2_ coverage indicates that these stronger interactions are limited to the primary high-affinity adsorption sites, whereas subsequent adsorption occurs at weaker physisorption sites. This trend highlights the challenge of maintaining sufficiently strong H_2_–framework interactions over a broad loading range, particularly for practical storage under near-ambient-temperature conditions.

Zhao et al. [179] focused on optimizing the solvent to improve the H_2_ uptake of UiO-66 [179]. Systematic synthesis using different solvents revealed that DMF plays a crucial role in obtaining phase-pure UiO-66. This solvent effect may be related to the ability of DMF to solvate both the amphiphilic dicarboxylate linker and polar Zr precursor species, thereby promoting the controlled assembly of Zr_6_ nodes and BDC linkers. In contrast, solvents with less suitable polarity and precursor-solvation ability resulted in amorphous products or UiO-66 contaminated with residual or polymerized H_2_BDC. UiO-66 synthesized in DMF at 50°C exhibited high crystallinity, with an *S*_BET_ of 1358 m^2^ g^−1^ and a *V*_p_ of 0.43 cm^3^ g^−1^, and achieved a H_2_ uptake of 3.35 wt% at 77 K and 1.8 MPa. Furthermore, grand canonical Monte Carlo simulations suggested that H_2_ molecules preferentially adsorb near the phenyl rings of the BDC linker rather than exclusively at the inorganic node, highlighting the contribution of the BDC linker to the local adsorption environment for H_2_ molecules. Ren et al. [180] reported a formic acid-modulated synthesis of UiO-66 to improve its crystallinity and H_2_ uptake [180]. Upon increasing the amount of formic acid, the *S*_BET_ value increased from 241 to 1367 m^2^ g^−1^, while the H_2_ uptake increased from 0.35 to 1.5 wt% at 77 K and 0.1 MPa.

**Figure 8.**
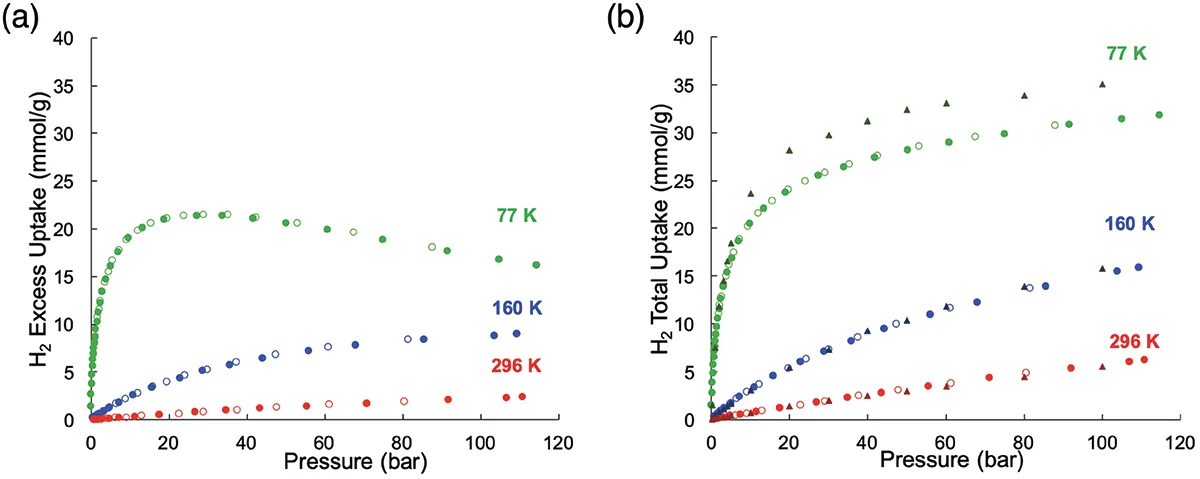
(a, b) temperature-dependent excess (a) and total (b) hydrogen adsorption (filled points) and desorption (open points) isotherms at 77 (green), 160 (blue), and 296 K (red). Filled triangles represent simulated isotherms for UiO-67 based on grand canonical Monte Carlo calculations. Reproduced with permission [97] 2018, American Chemical Society.

García-Holley et al. [97] reported a benchmark study of H_2_ storage in 14 representative MOFs under standardized temperature- and pressure-swing conditions to assess the H_2_ storage performance under tank-relevant operating conditions [97]. In this dataset, the temperature-dependent H_2_ uptake of UiO-67 at 77, 160, and 296 K was reported. UiO-67 was prepared using an optimized procedure. The obtained UiO-67 exhibited an *S*_BET_ value of 2360 m^2^ g^−1^ with *V*_p_ = 0.91 cm^3^ g^−1^. Temperature-dependent H_2_ adsorption and desorption studies showed a fully reversible behavior. Approximate maximum excess H_2_ uptakes were estimated by digitizing the reported plots, giving 22 mmol g^−1^ (4.4 wt%) at 77 K (~3 MPa), 9 mmol g^−1^ (1.8 wt%) at 160 K (11 MPa), and 2.5 mmol g^−1^ (0.5 wt%) at 296 K (11 MPa) (Figure 8(a)). Meanwhile, the corresponding total uptakes at 10 MPa, accounting for the contribution of compressed H_2_ in the *V*_p_, were 31.14 mmol g^−1^ (6.28 wt%), 29.29 mmol g^−1^ (5.90 wt%), and 5.74 mmol g^−1^ (1.16 wt%) at 77, 160, and 296 K, respectively (Figure 8(b)). The authors further calculated the deliverable capacity, which is defined as the difference between the total H_2_ uptake at 77 K and 10 MPa (charging) and that at 160 K and 0.5 MPa (discharging), in accordance with the tank design criteria. Under these conditions, UiO-67 delivered 6.0 wt% H_2_. On a volumetric basis, UiO-67 delivered 41 g L^−1^, corresponding to a mid-range value within the studied MOFs. It must be noted that the volumetric capacities were calculated by using monolithic density (*ρ* = 0.688 g cm^−3^) of UiO-67. Therefore, the practical performance may be lower, depending on the achievable packing density in a real tank system.

Notably, unlike UiO-66 and UiO-67, systematic H_2_ adsorption data for pristine UiO-68 remain scarce, although simulation studies have been reported [181]. One possible reason originates from the synthetic challenges, as the extended linker of UiO-68 (H_2_TPDC) often exhibits limited solubility in conventional solvothermal media. This may have hindered the reproducible crystal growth of UiO-68.

### 3.3. H_2_ storage in functionalized UiO-MOFs

**Figure 9.**
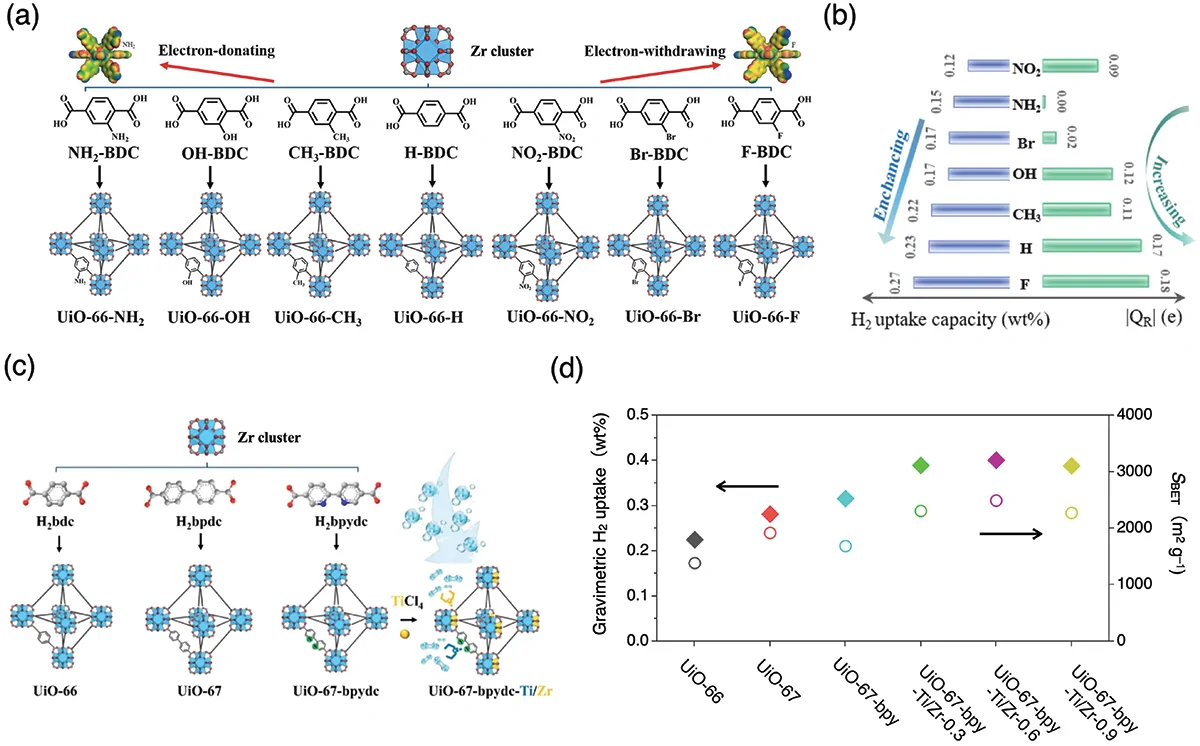
(a, b) Illustration of a series of UiO-66-X (X = H, CH_3_, NH_2_, OH, F, Br, and NO_2_) (a) and the relationship between the excess H_2_capacity of UiO-66-X at 298 K and absolute value of the average electrostatic potential-derived charge (|Q_R_|) (b). Reproduced with permission [182] 2026, Elsevier. (c) Schematic of the synthesis of UiO-66, UiO-67, UiO-67-bpydc, and UiO-67-bpydc-Ti/Zr. Reproduced with permission [183] 2025, American Chemical Society. (d) Plots of their excess H_2_uptake measured at 298 K and 10 MPa (left axis) and*S*_BET_values (right axis) for these UiO materials. Data extracted from ref. [183] and plotted by the authors.

Functional group engineering in MOFs has proven to be a powerful paradigm for modulating the interactions with H_2_. Recently, Wang et al. [182] systematically investigated the impact of linker functionalization on the hydrogen storage performance of UiO-66 derivatives [182]. Seven UiO-66-X frameworks (X = H, CH_3_, NH_2_, OH, F, Br, and NO_2_) were synthesized via a solvothermal method using X-substituted BDC linkers (Figure 9(a)). N_2_ sorption measurements showed that functionalization generally reduced *S*_BET_ and *V*_p_ compared with pristine UiO-66 (1386 m^2^ g^−1^ and 0.56 cm^3^ g^−1^, respectively) due to steric occupation of pore spaces. Consequently, all the derivatives exhibited lower H_2_ uptake than pristine UiO-66 (2.11 wt%) at 77 K and 5 MPa. However, under ambient conditions (298 K and 10 MPa), UiO-66-F showed the highest uptake of 0.27 wt%, corresponding to a 22% enhancement over pristine UiO-66 (0.22 wt%), whereas the other derivatives showed an inferior performance. As shown in Figure 9(b), the H_2_ uptake generally correlates with the magnitude of the average electrostatic potential-derived charge of the functional group (|Q_R_|), suggesting that the extent of charge redistribution plays a key role in modulating the interaction between the framework and H_2_. In this context, a larger |Q_R_| generally corresponds to higher H_2_ uptake, although |Q_R_| alone does not fully account for the behavior of the NO_2_-substituted derivative, indicating that additional factors such as the size of the functional group should also be considered. Therefore, |Q_R_| should be regarded as a useful, albeit simplified, indicator of the electronic effect, while the overall adsorption behavior is still influenced by the detailed local electronic environment of each substituent.

Building on ligand functionalization, Su et al. [183] systematically investigated how ligand length, types of functional groups, and metal node variations affect H_2_ storage performance in the UiO-type MOFs [183]. They synthesized UiO-66, UiO-67, a bipyridine-functionalized UiO-67 analog (UiO-67bpy), and a Ti-substituted UiO-67bpy (UiO-67-bpy-Ti/Zr-*n*) following a reported procedure with minor modifications (Figure 9(c)). For UiO-67-bpy-Ti/Zr-*n*, *n* denotes the molar fraction of Ti relative to Zr in the metal nodes, quantified using inductively coupled plasma optical emission spectrometry (ICP-OES), and *n* = 0.3, 0.6, or 0.9. H_2_ adsorption measurements revealed clear structure – property trends across these three values (Figure 9(d)). Specifically at 298 K and 10 MPa, extending the linker from UiO-66 to UiO-67 increased excess H_2_ uptake from 0.23 to 0.28 wt%, consistent with the enlarged porosity associated with the longer ligand. Compared with UiO-67, UiO-67-bpy exhibited improved H_2_ storage from 0.28 to 0.32 wt%, despite its lower surface area. This result highlights the contribution of nitrogen-containing functional groups to enhanced H_2_ physisorption. Furthermore, UiO-67-bpy-Ti/Zr-0.6 exhibited the highest excess H_2_ uptake of 0.40 wt%. X-ray photoelectron spectroscopy (XPS) and density functional theory (DFT) analyses suggested that Ti doping induces a local ‘strong-Zr, weak-Ti, negative-O’ potential gradient that enhances H_2_ polarization and stabilizes physisorption.

It is important to note that although functional-group engineering has made Δ*H* more negative, reaching values of approximately −15 to −20 kJ mol^−1^, this does not necessarily translate into higher adsorption capacity. The entropic penalty, which is often overlooked, plays a significant role [184]. As H_2_ molecules become more strongly bound to functional sites, the loss of translational and rotational freedom can offset the enthalpic benefits at 298 K. Consequently, a more negative Δ*H* is often accompanied by a significant entropy-driven compensation (enthalpy – entropy compensation) effect, and the net thermodynamic driving force should be evaluated in terms of the Gibbs free energy, Δ*G* = Δ*H* − *T*Δ*S*. Future research should therefore evaluate Δ*H*, Δ*S*, and Δ*G* together to provide a more complete thermodynamic understanding of hydrogen adsorption in functionalized MOFs.

Beyond simple linker substitution, various modification strategies, including cross-linking [185,186], mixed-ligand design [187], and composite formation [188–190], have been explored in UiO-66 to tune its hydrogen adsorption properties. Collectively, these results demonstrate that synergistic tuning of the linker length, functional groups, and metal-node composition enables the optimization of H_2_ physisorption beyond simple surface-area effects.

### 3.4. H_2_ storage in compressed UiO-66 and UiO-67

Considering potential applications, MOF powders must be packed into confined tank volumes, making the volumetric H_2_ capacity as critical as the gravimetric capacity. However, their inherently low bulk density typically imposes a significant limitation on the volumetric H_2_ capacity. Bambalaza et al. [94] reported the effects of high-pressure compaction on the gravimetric and volumetric H_2_ uptake of UiO-66 [94]. The prepared UiO-66 was systematically compacted into pellets under pressures up to 665 MPa. Remarkably, powder XRD (PXRD) analysis confirmed that the UiO-66 framework essentially retained its crystal structure upon compaction. N_2_ sorption measurements showed that the *S*_BET_ value of the compacted UiO-66 pellet was 1707 m^2^ g^−1^, which was approximately 98% of its original *S*_BET_, with a modest reduction in *V*_p_ owing to the elimination of interparticle voids. At 77 K and 10 MPa, the total gravimetric H_2_ uptake of the compacted UiO-66 pellet reached 5.1 wt%, which is essentially identical to that of the powder form (5.0 wt%). This result is in clear contrast to most reports on MOF compaction, which often causes surface area loss and reduces gravimetric uptake. The authors concluded that this mechanical robustness arises from the high minimum shear modulus of UiO-66 (~13.7 GPa), which is significantly higher than those of typical MOFs, such as ZIF-8 (~2.7 GPa) and MIL-53(Ga) (~0.16 GPa). Moreover, the packing density dramatically increased from ~0.57 to 1.45 g cm^−3^ upon compaction, resulting in a substantial enhancement in volumetric H_2_ capacity. For instance, at 77 K and 10 MPa, the total volumetric H_2_ capacity calculated using the packing density (1.45 g cm^−3^) reached 74 g L^−1^, which was more than two-fold higher than that of the powder form (29 g L^−1^). Later, the same authors further demonstrated that retention of hydroxylated Zr_6_O_4_(OH)_4_ nodes is important not only for enhancing H_2_ uptake, in agreement with the findings of Chavan et al. [184], but also for preserving the mechanical robustness of UiO-66 under high-pressure compaction [191].

**Figure 10.**
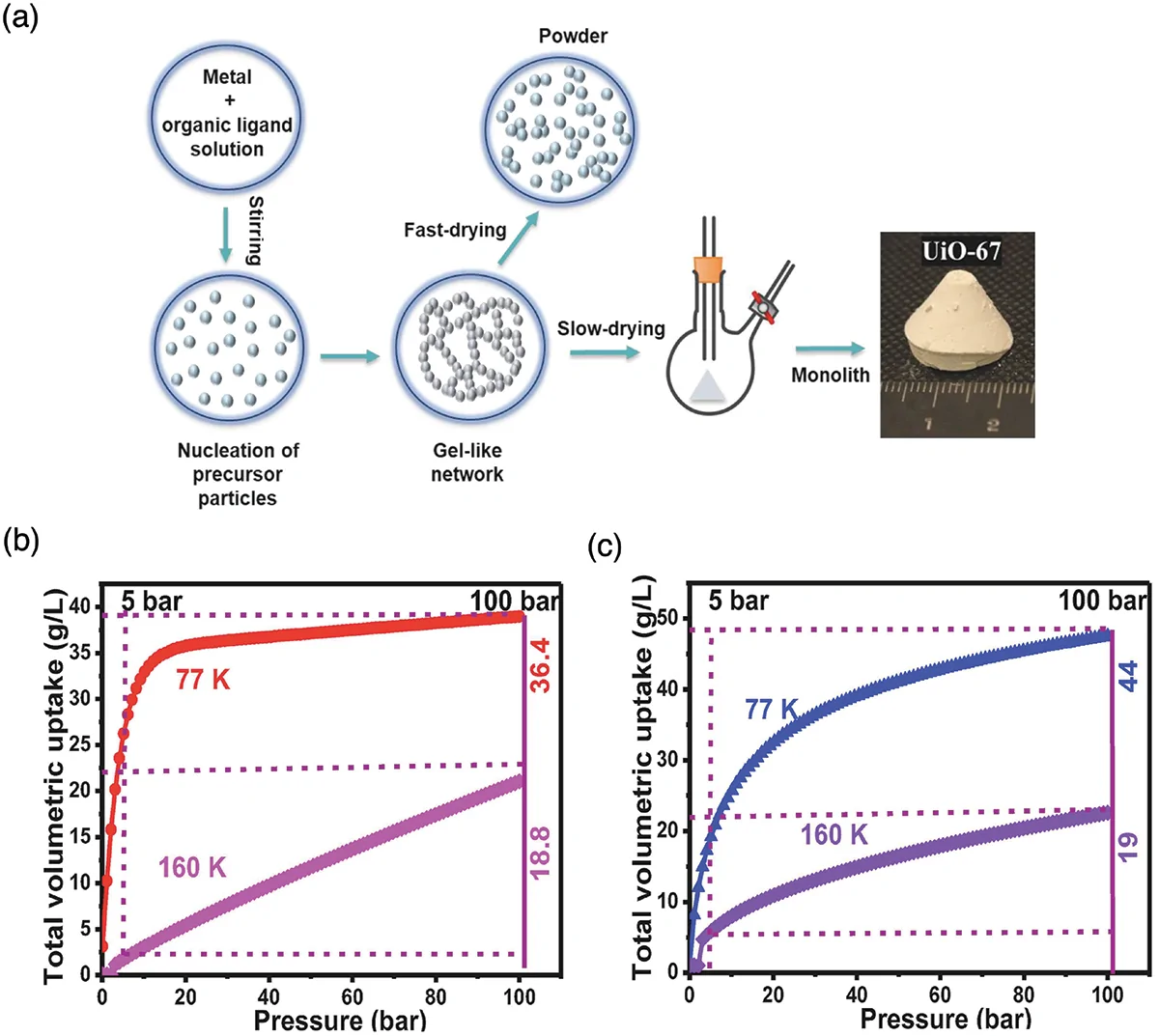
(a) Schematic representation of monolithic and powder MOF synthesis. (b, c) Total volumetric H_2_ storage at 77 and 160 K for _powd_UiO-67 (b) and _mono_UiO-67 (c) obtained using packing density. Reproduced with permission [95] 2025, Elsevier.

As an alternative densification approach to enhance the H_2_ volumetric capacity, Murugavel et al. [95] reported the densification of UiO-67 into binder-free monolithic structures via a sol – gel synthesis approach. Unlike conventional mechanical compaction, this strategy enabled the formation of high-density monolithic structures while preserving the intrinsic porosity of the framework. To yield high-density monolithic UiO-67 (_mono_UiO-67) and the powder form UiO-67 (_powd_UiO-67), the as-synthesized and washed UiO-67 gel-like product was divided into two portions: one portion was dried at room temperature to yield _mono_UiO-67, whereas the other was directly thermally activated to produce _powd_UiO-67 (Figure 10(a)). The resulting _mono_UiO-67 exhibited a bulk density (*ρ*_bulk_) of 0.68 g cm^−3^, which is significantly higher than that of _powd_UiO-67 (*ρ*_bulk_ = 0.155 g cm^−3^). Despite this large difference in *ρ*_bulk_, _mono_UiO-67 retained most of the textural and structural features of the powder form, showing only a modest decrease in *S*_BET_ from 2513 to 2303 m^2^ g^−1^ and in *V*_p_ from 0.81 to 0.73 cm^3^ g^−1^. H_2_ adsorption measurements revealed that the gravimetric uptake of _mono_UiO-67 slightly decreased upon densification, owing to a modest reduction in the surface area. By contrast, the volumetric uptake increased to ~48 g L^−1^ at 77 K and 10 MPa, compared with ~37 g L^−1^ for _powd_UiO-67. This enhancement was mainly attributed to the marked increase in *ρ*_bulk_ and the resulting increase in the volumetric hydrogen capacity of the monolithic sample. Furthermore, under temperature – pressure swing (TPS) conditions (charging at 77 K and 10 MPa, discharging at 160 K and 0.5 MPa), the delivered capacity of _mono_UiO-67 reached approximately 44 g L^−1^, exceeding that of _powd_UiO-67 (36 g L^−1^) (Figure 10(b,c)). These results highlight that densification into monolithic architectures is an effective strategy for enhancing the practical volumetric hydrogen storage of UiO-type MOFs, even when a slight reduction in gravimetric uptake occurs.

## 4. UiO-type MOFs as chemisorption-assisted hydrogen storage

While physisorption in UiO-type MOFs provides a reversible adsorption mechanism, its practical hydrogen storage performance is fundamentally limited by the weak H_2_–MOF interaction (Δ*H* ≈ −5 kJ mol^−1^). Volumetric and gravimetric uptake data indicate that these materials are already approaching their maximum storage capacities under typical high-pressure measurement conditions, particularly at room temperature. These limitations highlight the need for alternative or complementary strategies, such as spillover-assisted storage, chemisorption-assisted storage, and nanoconfined hydride storage, to further enhance hydrogen storage performance. In this section, we first summarize the fundamental concepts of spillover-assisted and hydride-based hydrogen storage, along with representative studies using UiO-type MOFs.

### 4.1. Spillover-assisted hydrogen storage on UiO-type MOFs

#### 4.1.1. Hydrogen spillover

Hydrogen spillover, where H_2_ dissociates into atomic hydrogen and migrates onto the host surface, has attracted significant attention as a strategy for generating and delivering reactive hydrogen species. Historically, hydrogen spillover has been primarily discussed in the context of heterogeneous catalysis since the 1960s [192–196], whereas its extension to hydrogen storage in porous materials has been developed more recently [197–201]. Hydrogen spillover typically requires a dissociation catalyst such as Pt- or Pd-based nanoparticles. Accordingly, MOFs serve as ideal model platforms for spillover studies, enabling the controlled placement of dissociation catalysts while offering hydrogen-accepting surfaces and transport pathways. Although hydrogen spillover has been widely investigated, its quantitative contribution to hydrogen storage remains controversial because of reproducibility issues and the difficulty of directly identifying mobile hydrogen species [202]. Among the various MOFs, UiO-type MOFs are robust hosts that can tolerate postsynthetic modifications and nanoparticle incorporation. Based on this background, this section summarizes the recent progress of spillover-assisted hydrogen storage in UiO-type MOFs.

#### 4.1.2. Room-temperature hydrogen storage using noble metal nanoparticles

**Figure 11.**
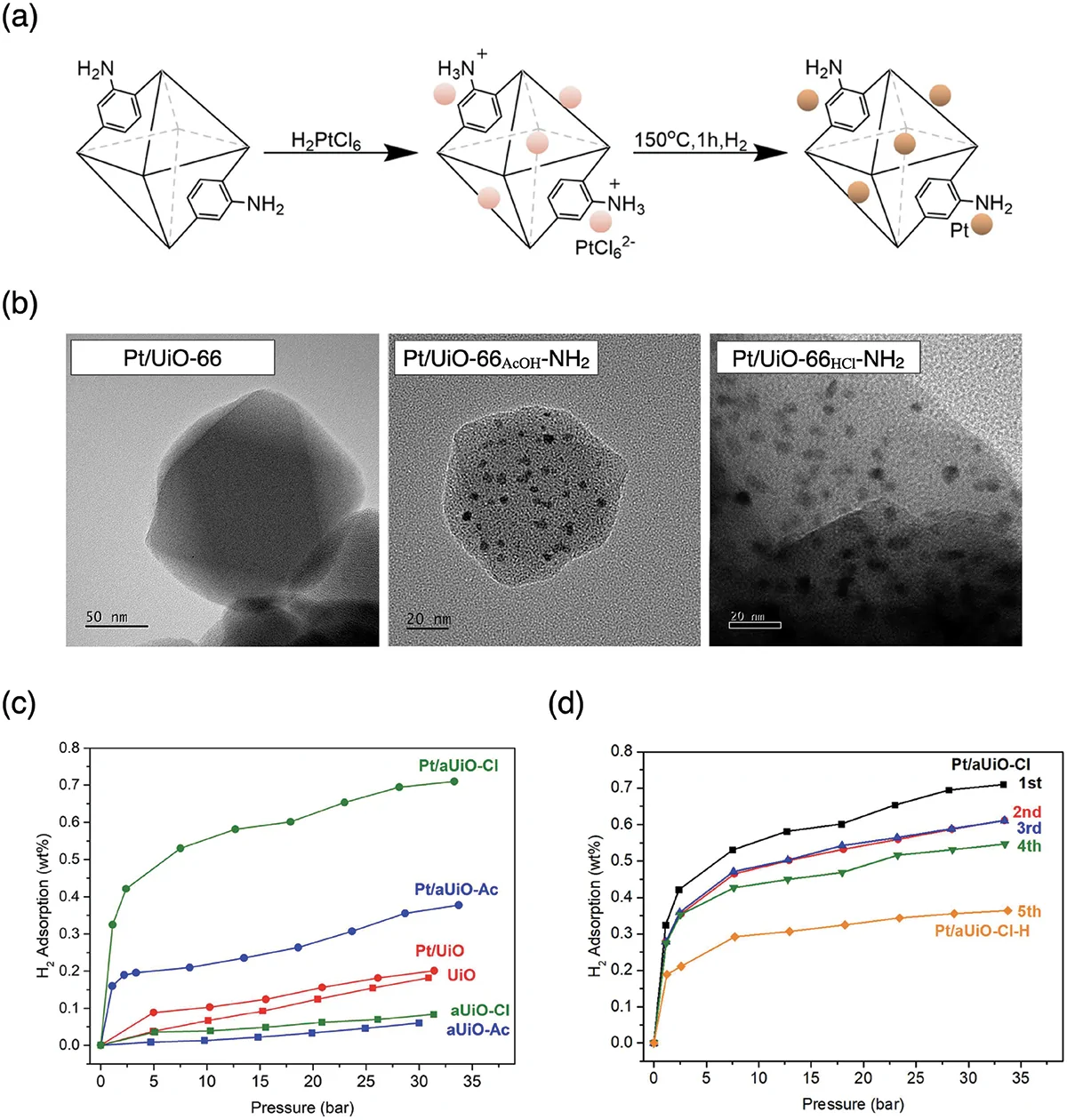
(a) Schematic of the synthesis of Pt/UiO-NH_2_ with the assistance of amino decoration to anchor platinum precursors. (b) FE-TEM images of Pt/UiO-66 (left), Pt/UiO- 66_AcOH-_NH_2_ (middle), and Pt/UiO-66_HCl-_NH_2_ (right). (c) Hydrogen adsorption isotherms of pure MOFs (UiO-66, UiO-66_AcOH-_NH_2_, and UiO-66_HCl-_NH_2_) and Pt-doped MOFs (Pt/UiO-66_AcOH-_NH_2_ and Pt/UiO-66_HCl-_NH_2_) at 30 °C up to 3.5 MPa H_2_. (d) Hydrogen adsorption cycling test of Pt/UiO-66_HCl-_NH_2_ at 30 °C up to 3.5 MPa H_2_. Reproduced with permission [203] 2021, American Chemical Society.

Although room-temperature hydrogen uptake is essential for practical hydrogen-storage applications, the physisorption of H_2_ onto porous adsorbents is typically weak at ambient temperatures. Kang et al. [203] addressed this limitation using a spillover strategy in UiO-66 and its amino-functionalized analog (UiO-66-NH_2_) as the host framework [203]. The UiO-66-NH_2_ samples were synthesized with different acid modulators (AcOH or HCl) and denoted as UiO-66_AcOH_-NH_2_ and UiO-66_HCl_-NH_2_, respectively. Pt decoration was carried out through the methanol-phase impregnation of the MOFs (UiO-66, UiO-66_AcOH_-NH_2_, and UiO-66_HCl_-NH_2_) with an H_2_PtCl_6_ precursor, followed by drying and H_2_ reduction to form Pt nanoparticles (150°C, 1 h) (Figure 11(a)). The amino groups in UiO-66-NH_2_ were expected to anchor Pt anions, thereby retaining the Pt species during reduction and leading to well-dispersed Pt nanoparticles (Figure 11(b)).

H_2_ adsorption isotherms at 30°C revealed that pristine UiO-66 and its amino-functionalized derivatives exhibited linear physisorption behavior, reaching around 0.06–0.18 wt% at 3.0 MPa and 30°C. By contrast, Pt-decorated UiO-66-NH_2_ (Pt/UiO-66_AcOH_-NH_2_ and Pt/UiO-66_HCl_-NH_2_) exhibited a pronounced enhancement in hydrogen uptake (Figure 11(c)). Among them, Pt/UiO-66_HCl_-NH_2_ showed the highest H_2_ uptake, reaching 0.71 wt% at approximately 3.3 MPa and 30°C. This enhancement was attributed to the hydrogen spillover effect, in which Pt nanoparticles catalytically dissociate H_2_ molecules, and the resulting hydrogen species migrate onto the UiO-66-NH_2_ framework. XPS analysis suggested that the spillover hydrogen species interact with, or hydrogenate, carboxylate groups within the framework. The modulator-controlled MOF particle size and Pt nanoparticle dispersion were also considered important factors facilitating the spillover process. Importantly, cycling measurements were conducted to evaluate reversibility under practical conditions. Cyclic tests of Pt/UiO-66_HCl_-NH_2_ at 30°C showed that the initial uptake (0.71 wt% at ~3.3 MPa) gradually decreased to 0.36 wt% after five cycles (Figure 11(d)). TEM images indicated agglomeration of the Pt nanoparticles after cycling. Additionally, the O 1s XPS spectra showed an increased hydroxyl component, suggesting the partial loss or occupation of hydrogen-accepting sites.

Notably, such a significant decrease in hydrogen uptake over only a few cycles is not unique to this material. Rather, it reflects a broader limitation frequently observed in spillover-mediated hydrogen storage systems, in which repeated cycling can lead to degradation of active sites and a consequent reduction in storage capacity [202,204,205]. Although UiO-type MOFs generally maintain their stability under high-pressure hydrogen cycling, active hydrogen species generated during spillover may induce structural degradation at defect sites. Such processes may result in irreversible structural changes, including framework unzipping [206], particularly in regions containing a high concentration of defects.

Wang et al. [207] reported the enhancement of room-temperature hydrogen uptake by Pd-decorated UiO-66-NH_2_ (Pd/UiO-66-NH_2_) [207]. In their system, Pd nanoparticles were introduced via the chemical reduction of PdCl_2_ using NaBH_4_ in an aqueous UiO-66-NH_2_ suspension. Hydrogen adsorption measured at 298 K showed a markedly increased uptake, reaching ~3.7 wt% at 20 MPa. Notably, the authors proposed that the ultrathin carbon layer observed at the Pd–MOF interface could lower the energy barrier for hydrogen migration. However, cyclability was not reported in this study, so the durability of the enhanced uptake remains unclear.

#### 4.1.3. Room-temperature hydrogen storage using non-noble metal nanoparticles

**Figure 12.**
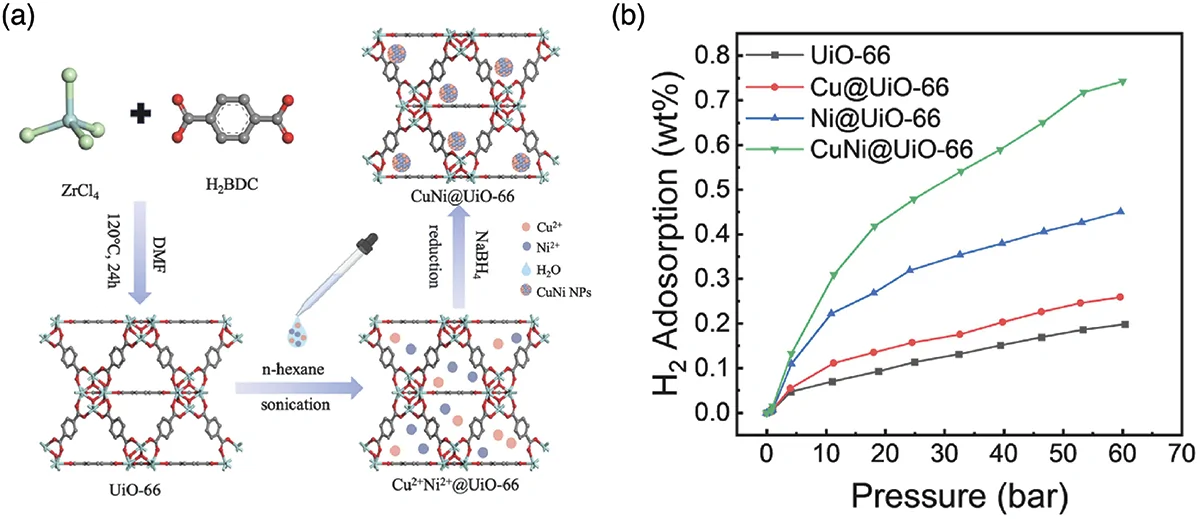
(a) Schematic of the synthesis process for the CuNi@UiO-66 composite. (b) Hydrogen adsorption isotherms of UiO-66, Cu@UiO-66, Ni@UiO-66, and CuNi@UiO-66 at 298 K and 6.0 MPa. Reproduced with permission [208] 2024, Elsevier.

In addition to noble metals, Liu et al. [208] demonstrated that inexpensive transition-metal nanoparticles can also induce an efficient spillover effect in UiO-66. Cu, Ni, or bimetallic CuNi nanoparticles were incorporated into UiO-66 via a double-solvent impregnation of UiO-66 in *n*-hexane with an aqueous solution of CuCl_2_·2 H_2_O and/or NiCl_2_·6 H_2_O, followed by NaBH_4_ reduction, yielding Cu@UiO-66, Ni@UiO-66, and CuNi@UiO-66 (Figure 12(a)). Hydrogen adsorption isotherms at 298 K showed that pristine UiO-66 exhibited near-linear physisorption with a maximum uptake of 0.20 wt% at 6.0 MPa, whereas CuNi@UiO-66 displayed a pronounced enhancement, reaching 0.74 wt% at 6.0 MPa (Figure 12(b)). Notably, the authors concluded that the spillover efficiency of CuNi@UiO-66 was comparable to those of previously reported Pd- or Pt-modified MOFs. DFT calculations predicted that H_2_ adsorbs more favorably on the CuNi surface and that the energy barrier for H–H bond dissociation is lower than that on monometallic Cu or Ni, which supports the enhanced hydrogen uptake observed for CuNi@UiO-66. However, cycling performance was not reported in this study. In any case, an important challenge in spillover-based hydrogen storage is the design of systems that enable atomic hydrogen to recombine and desorb through associative desorption.

### 4.2. Tuning thermodynamics and kinetics of hydrides via nanoconfinement in UiO-type MOFs

#### 4.2.1. Nanoconfinement

Hydrides, in which hydrogen is chemically bound to metal centers, are an important class of solid-state hydrogen carriers that offer high volumetric hydrogen density and inherent safety advantages [209–212]. However, bulk metal hydrides typically require elevated temperatures for dehydrogenation and high H_2_ pressures for rehydrogenation, rendering onboard utilization and regeneration unfeasible. To address these limitations, reducing the size of the metal hydrides has become a well-established strategy for engineering the thermodynamics, kinetics, and reversibility of hydrogen chemisorption [168,213–215]. In this context, MOFs provide an attractive platform for hydride species because of their well-defined pore architectures and chemically tunable internal surfaces [216–218]. Herein, we focus on the use of UiO-type MOFs as host frameworks to probe the effects of nanoconfinement on hydride materials toward the development of practical hydrogen-storage systems.

#### 4.2.2. Nanoconfined metal borohydride

**Figure 13.**
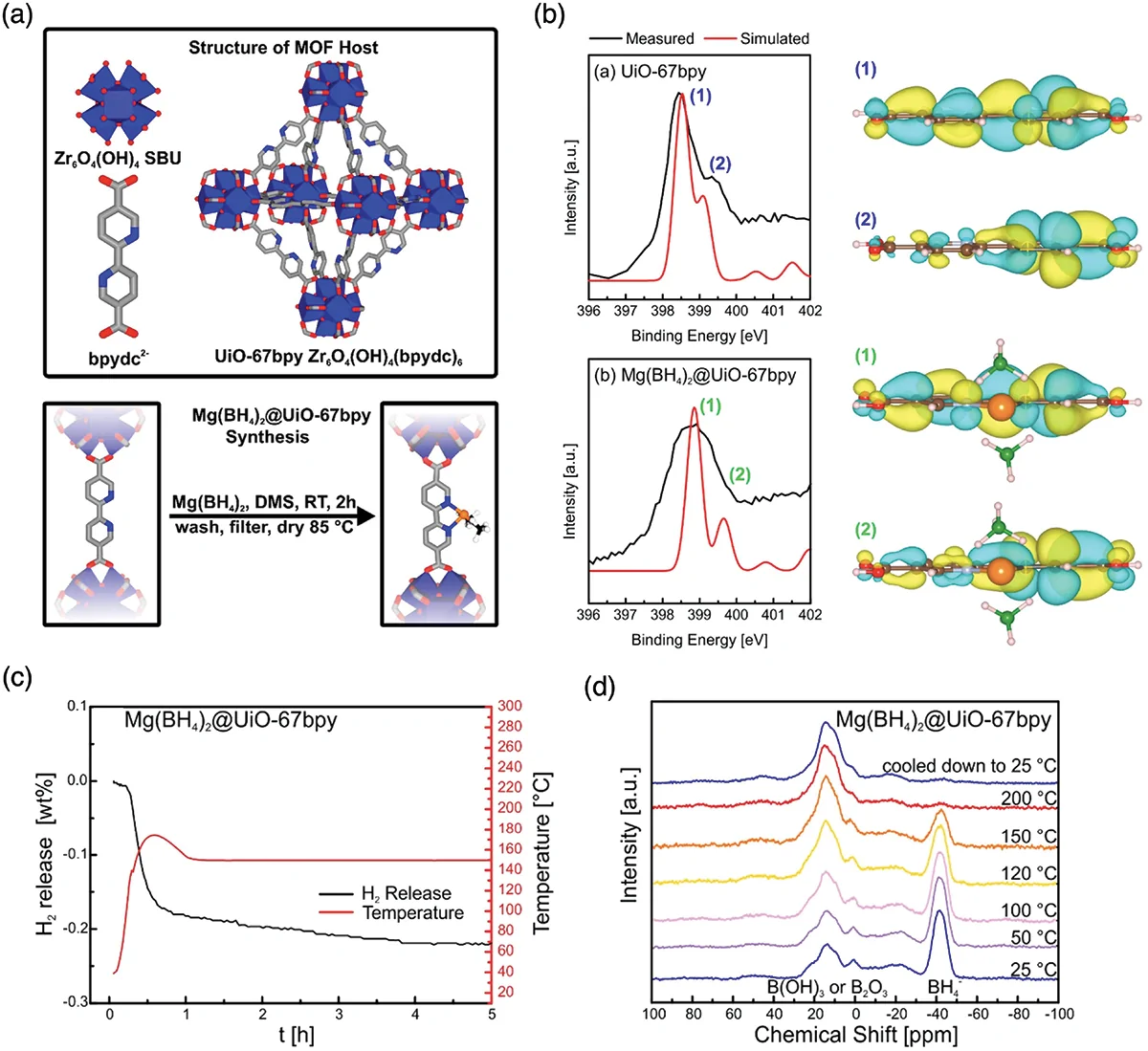
(a) Schematics of the organic and inorganic building blocks of UiO-67bpy and the octahedral pore (top) and the installation of Mg(BH_4_)_2_ at the bipyridine sites of UiO-67bpy (bottom). (b) N K-edge X-ray absorption spectra of UiO-67bpy (top) and Mg(BH_4_)_2_@UiO-67bpy (bottom). The black and red solid lines represent the experimental results and simulated spectra, respectively. (1) and (2) denote the 1^st^ and 2^nd^ excited states, and corresponding charge densities are shown in the right panel. (c) Sieverts measurement of Mg(BH_4_)_2_@UiO-67bpy used to quantify the amount of gas released at 150°C. The red curve represents the temperature profile, and the black curve represented the wt% of released hydrogen. (d) Variable-temperature ^11^B-MAS NMR spectra of Mg(BH_4_)_2_@UiO-67bpy. Reproduced with permission [219] 2025, American Chemical Society.

Inside MOFs, metal hydrides can be stabilized as nanocrystals or nanoclusters, thereby inhibiting their agglomeration. To better understand the limitations and stabilization mechanisms of nanoclustering, Schneemann et al. [219] investigated metal hydride nanoconfinement using UiO-67 consisting of 2,2’-bipyridine-5,5’-dicarboxylate (UiO-67bpy) as a 3D-porous framework [219]. Owing to the electron-rich bipyridine nitrogen sites capable of anchoring electron-deficient metal species through donor – acceptor interactions, magnesium borohydride (Mg(BH_4_)_2_) was selected as a model hydride because of its high gravimetric hydrogen capacity (14.9 wt %) [220–223]. Mg(BH_4_)_2_ incorporated into UiO-67bpy (Mg(BH_4_)_2_@UiO-67bpy) was prepared using a facile solvent-impregnation method (Figure 13(a)). Structural and spectroscopic analyses revealed that Mg(BH_4_)_2_ was confined within the pores in a highly dispersed, nearly molecularly dispersed state rather than as bulk crystalline domains. Importantly, N K-edge X-ray absorption spectroscopy showed a blue shift in the absorption edge from 398.54 eV for UiO-67bpy to 398.85 eV for Mg(BH_4_)_2_@UiO-67bpy, indicating charge transfer from the bipyridine N atoms to Mg (II) upon coordination at the pyridinic nitrogen sites (Figure 13(b)). Hydrogen-release measurements further showed that Mg(BH_4_)_2_@UiO-67bpy begins to desorb H_2_ at 120°C, whereas bulk Mg(BH_4_)_2_ typically desorbs H_2_ at 270–280°C (Figure 13(c)). This behavior was attributed to confinement-induced electronic modulation that weakens the B–H bonds and facilitates hydrogen release. Variable-temperature ^11^B magic-angle spinning NMR (^11^B MAS-NMR) further showed that Mg(BH_4_)_2_ confined within UiO-67bpy gradually decomposes upon heating, as the BH_4_^−^ signal decreased while signals assigned to B–O species increased (Figure 13(d)). Rehydrogenation was unsuccessful, likely owing to the formation of stable B–O species that limit reversibility. Therefore, designing systems capable of reversible hydrogen uptake after H_2_ release is of critical importance.

#### 4.2.3. Nanoconfined metastable metal hydride

**Figure 14.**
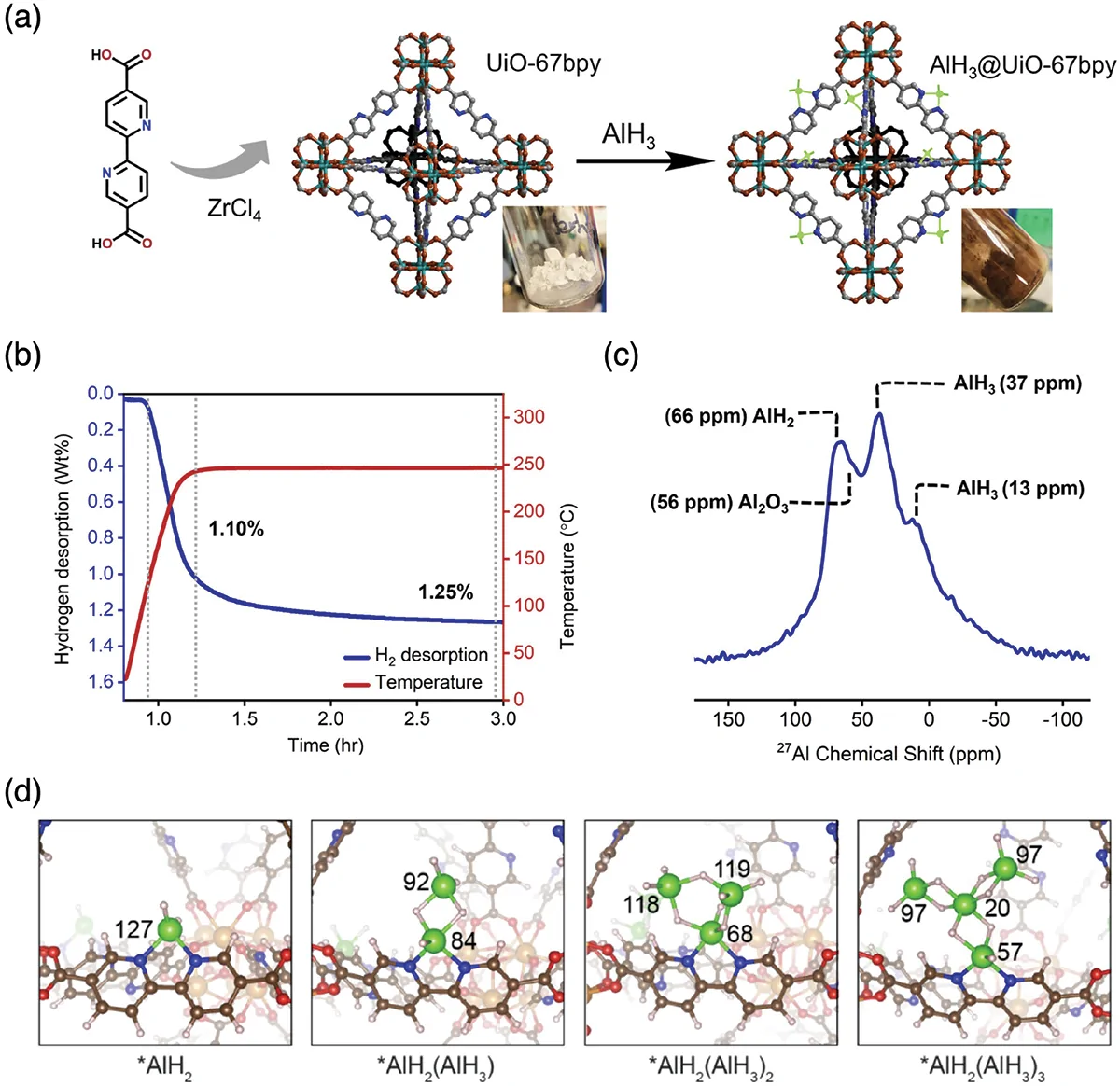
Synthesis and characterization of AlH_3_@UiO-67bpy. (a) Schematic of UiO-67bpy synthesis. Color changes observed upon infiltration with AlH_3_ are shown in the top right. (b) First cycle Sieverts measurements for AlH_3_@UiO-67bpy. (c) ^27^Al-MAS NMR profile of AlH_3_@UiO-67bpy. The three major ^27^Al signals at 13, 37, and 66 ppm correspond to AlH_3_, AlH_3_, and *AlH_2_, respectively. A small peak was found at 56 ppm for Al_2_O_3_. (d) DFT-optimized structures of complexed AlH_2_ bound by (AlH_3_)*_x_* (*x* = 0–3). In the structural models, green and pale pink spheres represent Al and H, respectively. The predicted Al chemical shifts of all Al atoms are shown for each structure. Reproduced with permission [224] 2025, American chemical Society.

Furthermore, Shivanna et al. [224] extended this concept [219] to the stabilization of metastable metal hydrides (AlH_3_) [225–227], within the same UiO-67bpy framework (AlH_3_@UiO-67bpy) (Figure 14(a)). Hydrogen release measurements showed that AlH_3_@UiO-67bpy released H_2_ corresponding to 1.10 wt% within 30 min at 250°C, reaching 1.25 wt% after an additional 2 h (Figure 14(b)). Notably, rehydrogenation at 70 MPa H_2_ and 60°C for 72 h enabled partial reversibility, whereas an analogous composite prepared from the unfunctionalized UiO-67 framework (AlH_3_@UiO-67) showed no detectable reversibility under the same conditions. ^27^Al magic angle spinning nuclear magnetic resonance (^27^Al MAS NMR) spectroscopy showed three distinct Al chemical environments at 13 and 37 ppm, assigned to AlH_3_ species, along with an additional signal at 66 ppm attributed to AlH_2_ (Figure 14(c)). DFT calculations predicted that AlH_3_ dissociates upon coordination to the bipyridine site and subsequently undergoes hydrogen spillover to the *β*-carbon of the linker, forming AlH_2_ directly coordinated to the bipyridine sites (denoted as *AlH_2_). This dissociation process is derived from the nearly full electron (0.82e^–^) transfer from AlH_3_ to the bipyridine linker. Further calculations on the AlH_2_(AlH_3_)_*x*_ cluster models qualitatively supported these assignments, suggesting that the 66-ppm resonance corresponds to *AlH_2_, while the signals at 13 and 37 ppm arise from the AlH_3_ clusters anchored to *AlH_2_ (Figure 14(d)). Their findings provide a platform for tuning the thermodynamics and kinetics of metastable materials using UiO-67bpy.

#### 4.2.4. Nanoconfined chemical hydride

**Figure 15.**
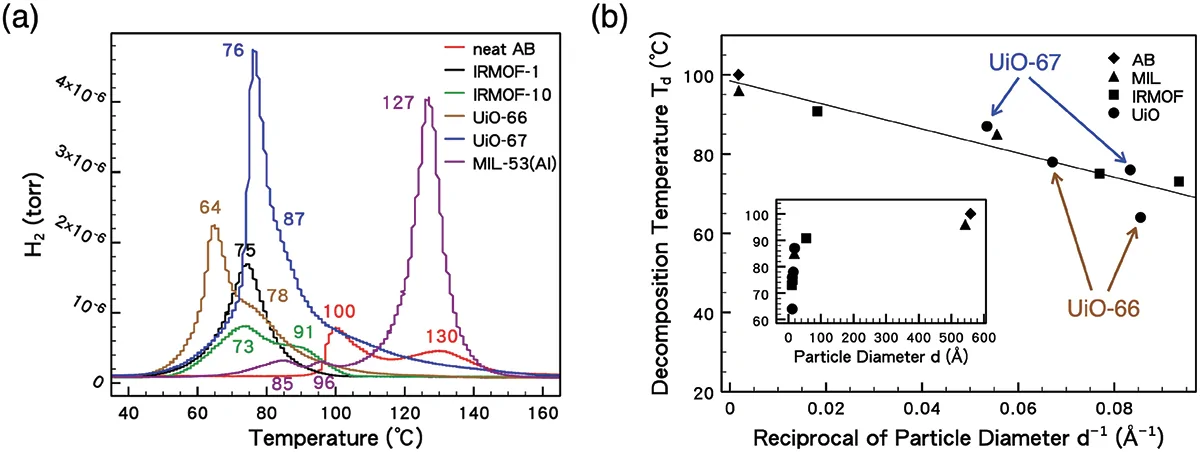
Temperature-programmed desorption with mass spectroscopy (TPD-MS) results for hydrogen desorption from AB (red) and AB in IRMOF-1 (black), IRMOF-10 (green), UiO-66 (red), UiO-67 (blue), and MIL-53(Al) (purple). AB decomposition peaked temperatures (*T*_d_) of neat AB (◆) and AB incorporated in MOFs of MIL MOFs (▲), IRMOFs (■), and UiO MOFs (●) vs. the reciprocal particle size (*d*) of corresponding hydrides. *T*_d_ vs. *d* is shown in the inset. Reproduced with permission [231] 2017, American Chemical Society.

This nanoconfinement strategy has also been applied to other chemical hydrides. Ammonia borane (AB: NH_3_BH_3_), which has a high hydrogen content (19.5 wt%) and is chemically stable at room temperature, has been extensively studied to lower its hydrogen-release temperature and suppress the formation of undesired byproducts [104,228–230]. Chung et al. [231] systematically studied the effect of nanoconfinement on AB using a series of MOFs, including UiO-66 and UiO-67, to elucidate the roles of pore size and porosity in hydrogen generation [231]. AB was incorporated into UiO-66 and UiO-67 using a solution-infiltration method, yielding AB@UiO-66 and AB@UiO-67, respectively. The PXRD pattern of AB@UiO-66 was almost identical to that of the neat MOF, with no apparent diffraction peaks from AB, indicating that highly dispersed or amorphous AB was confined within the smaller pores. By contrast, AB@UiO-67 exhibited weak additional diffraction signals attributable to AB, implying partial recrystallization in a larger pore environment than that of UiO-66. Temperature-programmed desorption mass spectrometry (TPD – MS) revealed that hydrogen (*m/z* = 2) release from AB confined in MOFs shifted to significantly lower temperatures compared to neat AB (100 and 113 °C). The decomposition temperature of AB decreased as the AB domain size decreased. Consistent with this trend, AB@UiO-66, which had the smallest pore size among the studied MOFs, exhibited the lowest H_2_ desorption temperatures (64 and 78 °C) (Figure 15).

## 5. Summary

This review summarized recent progress in hydrogen storage studies using UiO-type MOFs as robust and structurally tunable platforms. A summary of representative UiO-type MOFs and their reported H_2_ uptake values is provided in Table 2. For hydrogen adsorption, an ideal MOF would combine several demanding requirements: facile and scalable synthesis, high accessible surface area, and high framework robustness. In this context, UiO-type MOFs occupy a distinct position because they already offer relatively accessible synthesis, exceptional chemical and thermal robustness, and broad structural tunability. Beyond their promising potential as hydrogen adsorbents, UiO-type MOFs also provide well-defined platforms for systematically examining key structural and chemical factors that govern hydrogen storage behavior, including surface area, pore structure, linker functionality, framework densification, metal nanoparticle incorporation, and nanoconfined hydride species. Thus, UiO-type MOFs should be regarded not only as hydrogen adsorbents but also as model systems for understanding the structure – property relationships that govern solid-state hydrogen storage materials.

**Table 2.** List of selected UiO-type MOFs and reported H_2_ uptake values discussed in this review.

MOFs	S_BET_ (m^2^ g^−1^)	*V*_p_	Temp. (K)	Pres. (MPa)	Excess (wt%)	Ref.	Category
UiO-66	969	0.52	77	3.1	2.4	176	Physisorption
UiO-67	1877	0.95	77	3.8	4.6		
UiO-66	1434	–	77	6.0	4.2	177	
			298	6.0	0.5		
UiO-66	1358	0.43	77	1.8	3.35	178	
UiO-67	2360	0.91	77	3.0	4.4	97	
			160	11	1.8		
			298	11	0.5		
UiO-68-ANT	2950	1.17	77	3.0	5.3		
			160	11	2.3		
			298	11	0.6		
UiO-66	1386	0.56	298	10	0.23	182	Physisorption (linker functionalization)
			77	5.0	2.10		
UiO-66-CH_3_	949	0.4	298	10	0.22		
			77	5.0	2.10		
UiO-66-NH_2_	946	0.43	298	10	0.16		
			77	5.0	1.56		
UiO-66-OH	1077	0.43	298	10	0.17		
			77	5.0	1.74		
UiO-66-F	1023	0.54	298	10	0.27		
			77	5.0	1.89		
UiO-66-Br	748	0.31	298	10	0.17		
			77	5.0	1.41		
UiO-66-NO_2_	851	0.35	298	10	0.11		
			77	5.0	1.40		
UiO-66	1386	0.56	298	10	0.23	183	
			77	5.0	2.11		
UiO-67	1920	0.75	298	10	0.28		
			77	5.0	3.00		
UiO-67bpy	1688	0.71	298	10	0.32		
			77	5.0	3.50		
UiO-67bpy-Ti/Zr-0.3	2304	0.87	298	10	0.39		
			77	5.0	4.50		
UiO-67bpy-Ti/Zr-0.6	2488	0.96	298	10	0.40		
			77	5.0	4.53		
UiO-67bpy-Ti/Zr-0.9	2268	0.88	298	10	0.38		
			77	5.0	4.46		
UiO-66 powder	1737	0.96	77	10	2.1	94	Physisorption (compaction)
UiO-66 pellet	1707	0.81	77	10	2.7		
UiO-66 powder@80°C	1413	0.61	77	10	3.1	191	
UiO-66 pellet@80°C	1300	0.6	77	10	2.5		
UiO-67 powder	2513	0.81	298	10	0.51	95	
UiO-67 monolith	2303	0.73	298	10	0.42		
UiO-66	1390	0.61	303	3.0	0.18	203	Chemisorption (Spillover-assist)
UiO-66AcOH-NH_2_	1170	0.54	303	3.0	0.06		
UiO-66HCl-NH_2_	1070	0.44	303	3.0	0.08		
Pt/UiO-66AcOH-NH_2_	930	0.46	303	3.0	0.38		
Pt/UiO-66HCl-NH_2_	890	0.36	303	3.0	0.71		
Pd/UiO-66-NH_2_	–	–	298	2.0	3.7	207	
UiO-66	1231	0.6	298	6.0	0.2	208	
Cu@UiO-66	958	0.53	298	6.0	0.26		
Ni@UiO-66	1050	0.58	298	6.0	0.45		
CuNi@UiO-66	928	0.51	298	6.0	0.74		
Mg(BH_4_)_2_@UiO-67bpy	230–250	–	423	–	0.22	219	Chemical desorption (Hydride in UiO)
AlH_3_@UiO-67bpy	96	–	523	–	1.25	224	
AB@UiO-66	0.52	–	337, 351	–	1.08	231	
AB@UiO-67	0.95	–	349, 360	–	0.98		

For physisorption-based storage, the practical advantage of UiO-type MOFs becomes most apparent when their performance is evaluated under densification conditions. Many high-performing MOFs achieve large H_2_ capacities through maximizing surface area and pore volume, but their low bulk density and structural fragility can limit their use in confined tank volumes. In contrast, the reported compaction of UiO-66 and monolithic processing of UiO-67 are particularly important because they demonstrate that UiO frameworks can improve volumetric H_2_ capacity while largely preserving crystallinity, accessible porosity, and gravimetric uptake. As shown in Figure 5, for example, UiO-68-ANT shows a deliverable capacity close to that of MOF-177, indicating that extended-linker UiO frameworks can approach the volumetric deliverable performance of high-surface-area benchmark MOFs while offering the additional advantage of Zr-based framework robustness. In addition, monolithic UiO-67 exceeds monolithic HKUST-1, suggesting that open metal sites do not necessarily dominate high-pressure deliverable performance. Although open metal sites enhance H_2_ binding at low coverage, the capacity under swing conditions is largely governed by accessible pore volume, framework density, and porosity retention after densification.

Functionalization can enhance the intrinsic H_2_ adsorption ability of UiO-type MOFs, but its effect must be interpreted carefully. Polar functional groups, such as nitrogen-containing linkers and heterometallic nodes, can strengthen local H_2_ interactions, particularly under near-ambient conditions. However, functional groups also occupy pore volume and may reduce accessible surface area, which leads to reduce in total H_2_ capacity. It is also important to note that stronger H_2_ binding sites do not necessarily improve usable capacity at 298 K, because the entropic penalty associated with the localization of H_2_ can offset the enthalpic gain. This highlights the importance of evaluating Δ*H*, Δ*S*, and Δ*G* together, using variable-temperature adsorption measurements to clarify whether newly introduced binding sites truly improve the thermodynamic driving force for H_2_ adsorption.

Spillover-assisted storage and hydride nanoconfinement extend the role of UiO-type MOFs from passive adsorbents to platforms for controlling reactive hydrogen species. In both approaches, the central challenge is not simply to activate H_2_ or lower the hydrogen-release temperature. The activated hydrogen species or dehydrogenated products must remain sufficiently reversible during desorption or rehydrogenation, rather than being irreversibly trapped at the framework. For spillover systems, hydrogen-accepting sites should promote migration without irreversibly trapping hydrogen. For nanoconfined hydrides, the hydride domain size and local hydride – framework interface must be controlled because excessive dispersion or direct contact with oxygen-rich Zr nodes may promote irreversible B–O, Al–O, or framework-bound species. These studies highlight the importance of controlling not only the size of reactive hydrogen-storage domains but also their spatial relationship with Zr_6_ nodes, organic linkers, and metal nanoparticles.

Collectively, these findings show that UiO-type MOFs provide a robust and structurally tunable platform for studying hydrogen adsorption, spillover-assisted storage, and hydride nanoconfinement. Their hydrogen storage behavior is governed by multiple factors, including accessible porosity, framework density, adsorption-site chemistry, and the spatial arrangement of metal species or hydride domains within the framework.

## 6. Perspective

Future UiO-based hydrogen storage research should move toward spatially controlled framework engineering, taking advantage of the robustness and modular pore chemistry of UiO-type MOFs. For physisorption-based storage, a key design target is not simply to create stronger H_2_-binding sites, but to optimize the number, distribution, and local environment of polar sites within accessible pores. In this regard, mixed-linker strategies provide a practical route to tune the density and spatial distribution of functional sites via the incorporation of a controlled fraction of linkers bearing polar groups or metal-coordination sites, while maintaining the overall UiO framework.

Building on this site-density control, highly dispersed Li^+^ sites represent an attractive example of spatially designed H_2_-binding centers. Inspired by theoretical studies on alkali-metal-decorated porous materials [232–235], isolated Li^+^ sites could enhance H_2_ affinity through charge-induced dipole interactions without requiring bulky functional groups. In UiO-type MOFs, oxygen-containing or anionic linkers, such as those bearing pendant carboxylate, sulfonate, or phenoxide groups, could be introduced through mixed-linker design and subsequently used as anchoring sites for Li^+^ ions. This approach could allow Li^+^ sites to be spatially dispersed along the pore walls while preserving pore volume and molecular mobility. Importantly, the density of Li^+^ sites should be optimized rather than simply maximized, because excessive polar sites may reduce pore volume, decrease pore accessibility, or overly restrict the motion of adsorbed H_2_ molecules. Therefore, Li-decorated UiO frameworks should be evaluated in terms of the balance among adsorption enthalpy, adsorption entropy, Gibbs free energy, pore volume, molecular mobility, and deliverable capacity. At the same time, molecular-level binding-site design must be evaluated under practically relevant conditions. Densification tolerance alone is not sufficient for assessing the applicability of UiO-type MOFs as hydrogen adsorbents. Although UiO-type MOFs are widely recognized for their high chemical and thermal stability, repeated H_2_ adsorption – desorption cyclability has rarely been systematically examined. Therefore, future studies should report cycle-dependent H_2_ uptake, capacity-retention profiles, packing-density changes, and post-cycling structural and porosity characterization.

For chemisorption-related hydrogen storage, particularly spillover-assisted systems, future design should focus not only on H_2_ dissociation but also on controlling the migration pathway of dissociated hydrogen species. Catalytic metal sites and hydrogen-accepting regions should be positioned so that dissociated hydrogen can migrate through the framework without being irreversibly trapped at oxygen-rich Zr_6_ nodes or defect sites. In this regard, extended-linker structures, such as UiO-68-type frameworks, could be explored as future platforms for spatially separating catalytic metal sites from Zr–O/Zr–OH sites. Their larger pore spaces and longer linker regions may allow catalytic metal sites to be anchored on functionalized linkers, although such a design has not yet been systematically investigated for hydrogen storage. This spatial-design concept can also be extended to nanoconfined hydride systems. In these systems, future design should focus not just on confining hydride species within MOF pores, but on integrating confined hydride domains with nearby highly dispersed catalytic metal species in extended-linker UiO frameworks. For example, recent studies on borohydride-based nanocomposites have shown that nanoscale contact between hydride-derived domains and Ni catalytic clusters can substantially improve low-temperature hydrogenation and cycling reversibility [236,237]. These results suggest that hydride nanoconfinement should be coupled with spatially controlled catalytic H_2_ activation rather than treated only as a size-reduction strategy.

Furthermore, UiO-based composite architectures could provide another route to introduce confined hydrogen-storage environments that are difficult to achieve in UiO frameworks alone. One possible direction is the incorporation of porous hydride frameworks into UiO-based matrices. Schneemann et al. demonstrated Mg(BH_4_)_2_ nanoconfinement in UiO-67bpy, establishing a precedent for combining UiO-type frameworks with borohydride-based hydrogen storage materials. Building on this work, exploring whether porous hydride phases, such as γ-Mg(BH_4_)_2_, can be incorporated while preserving their intrinsic microporosity would be an attractive future direction. Such a design is particularly interesting because γ-Mg(BH_4_)_2_ can accommodate densely packed molecular H_2_ in its small pores, where H_2_ is stabilized via directional B–H^δ−^···H_2_ interactions with the hydridic pore surface [238,239]. If successful, this approach would expand hydride nanoconfinement beyond simple size reduction toward the design of porous hydride domains within robust UiO matrices. Another possible direction is the construction of UiO/2D-material composites based on the nanopump effect reported for stacked two-dimensional materials [240,241]. In this concept, interlayer spaces with approximately 6–7 Å can increase the local density of H_2_ through overlapping adsorption potentials from opposing two-dimensional surfaces. The role of UiO domains would be to serve as porous structural components that suppress dense restacking of the two-dimensional sheets and preserve H_2_-accessible pathways to these confined spaces, thereby improving adsorption kinetics without eliminating the local confinement effect.

Finally, advances in computational materials science, including high-throughput screening, molecular simulations, and machine-learning-assisted materials design, are expected to accelerate the discovery of promising linker chemistries, defect configurations, and catalytic compositions. The combination of data-driven materials discovery with experimentally guided synthesis may enable the rational development of UiO-type MOFs possessing balanced adsorption thermodynamics, rapid hydrogen transport, excellent cycling durability, and scalable synthesis.

Overall, future UiO-type MOF research should move beyond optimizing individual properties and focus on designing where and how H_2_-binding sites, catalytic metal species, and hydride domains are arranged within a robust porous framework. This direction is particularly suitable for UiO-type MOFs because their robust and modular frameworks allow physisorption-oriented binding-site design and chemisorption-related reactive-site design to be explored within the same structural platform. Such spatially controlled design will be essential for developing next-generation hydrogen storage materials that combine high usable capacity, reversibility, durability, and mechanistic tunability.
